# A Flexible Interpenetrated Diamondoid Metal–Organic Framework with Aromatic-Enriched Channels as a Preconcentrator for the Detection of Fluorinated Anesthetics

**DOI:** 10.1021/acs.chemmater.4c03221

**Published:** 2025-03-11

**Authors:** Danilo Marchetti, Nicolò Riboni, A. Ken Inge, Ocean Cheung, Mauro Gemmi, Enrico Dalcanale, Federica Bianchi, Chiara Massera, Alessandro Pedrini

**Affiliations:** † Department of Chemistry, Life Sciences and Environmental Sustainability, INSTM UdR Parma, University of Parma, Parco Area delle Scienze 17/A, Parma 43124, Italy; ‡ Department of Materials and Environmental Chemistry, Stockholm University, Frescativägen 8, Stockholm 10691, Sweden; § Division of Nanotechnology and Functional Materials, Department of Materials Science and Engineering, Ångström Laboratory, Uppsala University, Lägerhyddsvägen 1, Uppsala 75103, Sweden; ∥ Center for Materials Interfaces, Electron Crystallography, Istituto Italiano di Tecnologia, Viale Rinaldo Piaggio 34, Pontedera 56025, Italy

## Abstract

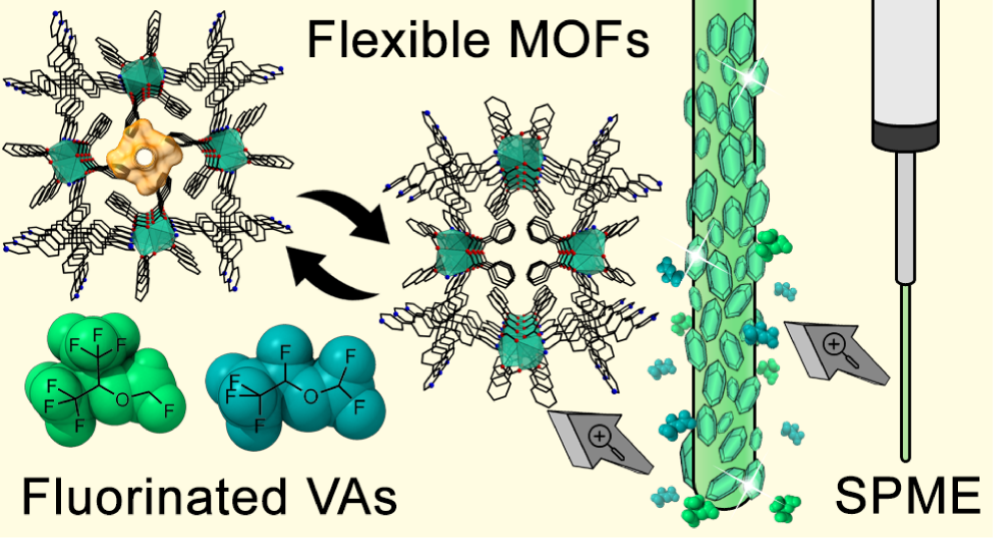

Flexible metal–organic frameworks (MOFs) are dynamic materials that combine long-range structural order with reversible stimulus-responsive phase transitions. In this study, we report the synthesis and characterization of two isoreticular flexible MOFs, **TPPM-CPW(Me)** and **TPPM-CPW(Ph)**, constructed by combining the ligand tetra-4-(4-pyridyl)phenylmethane (TPPM) with specific Cu(II) paddle-wheel (CPW) secondary building units (SBUs). These MOFs exhibit reversible transitions between open- and closed-pore forms triggered by external stimuli, such as temperature- and pressure-induced guest removal and uptake. The stability of these frameworks is influenced by the residual equatorial groups on the Cu(II) SBUs, with phenyl-functionalized **TPPM-CPW(Ph)** displaying dynamic behavior characteristic of third-generation soft porous crystals. Notably, **TPPM-CPW(Ph)** exhibited high adsorption affinity toward fluorinated guests, including SF_6_ and volatile anesthetics (VAs) such as desflurane and sevoflurane. This material, when used in solid-phase microextraction (SPME) as fiber coating for the preconcentration of these VAs in air, outperformed commercial CAR/PDMS fibers, underscoring the potential of these versatile flexible MOFs in addressing environmental challenges associated with the use of volatile fluorinated compounds.

## Introduction

Soft porous crystals are porous materials that possess both highly ordered networks and structural transformability.^1^ These crystalline solids couple long-range structural order with the capability to reversibly switch between states and feature at least one phase with permanent porosity. Most of the structural transitions of soft porous crystals are single-crystal-to-single-crystal (SCSC) transformations and involve framework rearrangements such as expansion, contraction, and breathing.^2^ In contrast to rigid porous frameworks, their intrinsic dynamic nature allows them to respond to external stimuli such as temperature, pressure, or guest molecules, making them suitable candidates for various technological applications. This behavior has been observed both in molecular crystals, such as supramolecular organic frameworks (SOFs),^3^ and in coordination frameworks like metal–organic frameworks (MOFs).^4,5^ Although rigid MOFs are still predominant compared to flexible MOFs, there are now several examples of MOFs endowed with structural flexibility.^6^ One of the most versatile classes of flexible MOFs is constituted of frameworks with interpenetrated structures, formed by two or more mechanically entangled nets that cannot be separated without breaking their bonds. In these MOFs, the degree of interpenetration, which can reach extraordinarily high values reported up to 54-fold interpenetration,^7^ is strictly correlated to the degree of porosity and therefore affects MOFs’ properties and functionality.^8,9^ As far as flexibility is concerned, the dynamic behavior of interpenetrated frameworks spans from the distortion of each individual net to the relative translation of multiple networks.^10^ This latter mechanism usually benefits from high degrees of interpenetration, although in some cases, an increase in the number of interpenetrated networks leads to the reduction of the space necessary for the dynamic response, thus suppressing MOF’s flexibility.^11^

Among flexible MOFs with interpenetrated structures, frameworks with diamondoid (**dia**) topology have demonstrated stimuli-responsive structural transformations induced by guest uptake and desorption.^12−21^ These materials are typically based on a tetrahedral inorganic secondary building unit (SBU) and linear ligands. On the contrary, organic struts with T_4_ symmetry are by far less exploited in this field. In particular, tetraphenylmethane (TPM) derivatives are rigid and versatile tectons that have been effectively applied to the preparation of porous networks of different nature.^3,22^ Specifically, MOFs constituted by TPM-based tectons endowed with carboxylate moieties^23−32^ or *N*-donor functional groups^33−40^ have been reported. To the best of our knowledge, no flexible MOFs based on these struts have been reported so far.

**Figure 1:**
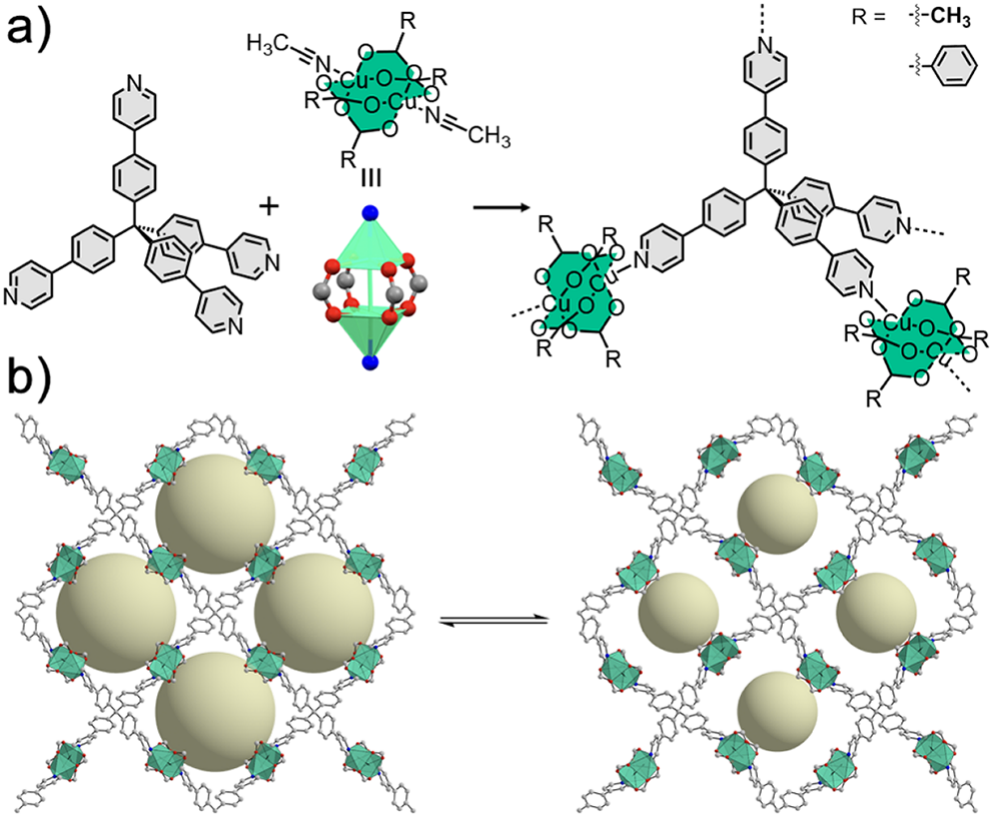
(a) General reaction between TPPM and [Cu_2_(RCO_2_)_4_(ACN)_2_] as a Cu(II) paddle-wheel source to obtain **TPPM-CPW(R)** (*R* = Me, Ph). (b) Example of a reversible crystal-to-crystal phase transition of a generic **TPPM-CPW(R)** between an open pore (left) and a closed pore (right) form. The R groups of the SBUs (which would point inside the channels) and hydrogen atoms have been omitted for clarity.

In this study, tetra-4-(4-pyridyl)phenylmethane (TPPM), already exploited in dynamic SOFs,^41^ was selected to achieve highly interpenetrated MOFs with diamond-like networks featuring dynamic behavior. In detail, two isoreticular TPPM-based MOFs were obtained by exploiting M_2_(CO_2_)_4_ SBUs, in which Cu(II) acts as the metal center (**TPPM-CPW(R)**, Figure 1a). These SBUs, known as Cu(II) paddle wheels (CPWs), usually give rise to 2D or 3D networks through polytopic carboxylate linkers. In this case, the apical positions of the Cu(II) centers were exploited to interact with the TPPM molecule, while two monotopic ligands, namely, acetate and benzoate, were selected as the SBU’s equatorial carboxylate ligands to afford **TPPM-CPW(Me)** and **TPPM-CPW(Ph)**, respectively. **TPPM-CPW(R)** undergoes a crystal-to-crystal phase transition between open-pore and closed-pore forms by applying external stimuli, such as temperature or pressure-induced removal of guest molecules embedded in their pores (Figure 1b). The residual groups connected to the COO^–^ units of the Cu(II) paddle wheels were found to be disposed facing the framework’s channels and played a key role in consolidating the MOF structure upon guest desorption. Finally, **TPPM-CPW(Ph)** textural parameters and absorption properties toward relevant gases were evaluated, and the material was tested as a preconcentrator for the adsorption of volatile fluorinated anesthetics by the solid-phase microextraction (SPME) technique.

## Experimental Details

### Materials

All commercial reagents and solvents were used as received. TPPM was synthesized following a previously reported procedure.^41^ The Cu(II) paddle wheel [Cu_2_(MeCO_2_)_4_(ACN)_2_] (ACN = acetonitrile) was prepared according to a procedure reported in the literature,^42^ while [Cu_2_(PhCO_2_)_4_(ACN)_2_] was obtained from an adapted one.^43^ These synthetic procedures are detailed in the Supporting Information.

#### Synthesis of **TPPM-CPW(Me)**

The synthesis was conducted by solubilizing the TPPM molecule (3.34 mg, 5.31 μmol) in 3.2 mL of a 2:1 CHCl_3_/*n*-BuOH solution and [Cu_2_(MeCO_2_)_4_(ACN)_2_]·ACN (5.12 mg, 10.75 μmol) in 2.8 mL of a 1:2 CHCl_3_/*n*-BuOH solution. The solution of TPPM was then placed into a glass tube with a Teflon screw cap, and then, the [Cu_2_(MeCO_2_)_4_(ACN)_2_]·ACN solution was carefully added to obtain two different layers. The reaction was then heated at 90 °C for 2 days, obtaining large crystals suitable for SC-XRD diffraction analysis.

#### Synthesis of **TPPM-CPW(Ph)**

Method A: The solvothermal synthesis was conducted by solubilizing the TPPM molecule (2.90 mg, 4.61 μmol) in 2.8 mL of a CHCl_3_/^i^PrOH 2:1 solution, benzoic acid (15.4 mg, 12.61 μmol) in 1.5 mL of a CHCl_3_/^i^PrOH 1:1 solution, and [Cu_2_(PhCO_2_)_4_(ACN)_2_]·ACN (6.97 mg, 9.62 μmol) in 2.8 mL of a CHCl_3_/^i^PrOH 1:2 solution. The solution of TPPM was then placed into a glass tube with a Teflon screw cap, and then, the benzoic acid solution, followed by the [Cu_2_(PhCO_2_)_4_(ACN)_2_]·ACN solution, was carefully added to obtain three different layers. The reaction was then heated at 50 °C for 3 days. The obtained green powder was rinsed with a 9:1 CHCl_3_/MeOH solution and dried under reduced pressure (5.1 mg, 60% yield). The product was characterized through scanning transmission electron microscopy imaging (HAADF-STEM), 3D electron diffraction (3D ED), and PXRD analysis. Method B: The mechanochemical synthesis was carried out by placing the TPPM molecule (29.5 mg, 46.9 μmol), [Cu_2_(PhCO_2_)_4_(ACN)_2_]·ACN (66.2 mg, 91.3 μmol) and 50 μL of benzyl alcohol into an agate jar with two 5 mm agate spheres. The mixture was ground in a Retsch mixer mill MM 400 operating at 20 Hz for 30 min. The ground product was left in an open container to evaporate the remaining traces of the LAG (Liquid-Assisted Grinding) additive. The obtained green powder was then washed with a 9:1 CHCl_3_/MeOH solution, dried under reduced pressure, and characterized through PXRD analysis (53.3 mg, 61% yield).

### Single Crystal X-ray Diffraction (SC-XRD)

The crystal structures of **TPPM-CPW(Me)** at two different temperatures were determined by X-ray diffraction on single crystals. Crystal data and experimental details for data collection and structure refinement are reported in Table S1. Intensity data and cell parameters were recorded at 200(2) and 300(2) K, respectively, for the contracted and expanded phase, on a Bruker D8 Venture Photon II diffractometer (CuKα radiation λ = 1.54178 Å). The raw frame data were processed using SAINT and SADABS to yield the reflection data files.^44^ The structures were solved by direct methods using the SHELXT program^45^ and refined on *F*_o_^2^ by full-matrix least-squares procedures using SHELXL-2018^46^ in the WinGX suite v.2021.2.^47^ All non-hydrogen atoms were refined with anisotropic atomic displacements. The hydrogen atoms were included in the refinement at idealized geometry and refined “riding” on the corresponding parent atoms. The weighting schemes used in the last cycle of refinement were *w* = 1/ [σ^2^*F*_o_^2^ + (0.1564*P*)^2^] and *w* = 1/[σ^2^*F*_o_^2^ + (0.2350*P*)^2^], where *p* = (*F*_o_^2^ + 2*F*_c_^2^)/3, for **TPPM-CPW(Me)** at 200 and 300 K, respectively. In both cases, the structures were subjected to the program SQUEEZE^48^ that detected a solvent-accessible void of 1424 and 2929 Å³ in the contracted and expanded phases, respectively, and an electron count per unit cell of 379 and 2929. This electron density is likely due to solvent molecules (chloroform and butanol). Their contribution to the diffraction pattern was removed, and the modified *F*_o_^2^ were written to a new HKL file. Since the residual electron density was smeared throughout the whole unit cell, no definite number of chloroform and butanol molecules was assigned nor included in the formula, formula weight, calculated density, μ, and F(000).

### 3D Electron Diffraction (3D ED) and TEM Analysis

Scanning transmission electron microscopy imaging and 3D electron diffraction were carried out on a Zeiss Libra 120 transmission electron microscope, equipped with a LaB_6_ thermionic source operating at 120 kV (λ = 0.0335 Å) and a Timepix single-electron detector by ASI for collecting diffraction patterns in low dose mode. 3D electron diffraction data were collected on single nanocrystals in nanodiffraction mode with a parallel electron beam 150 nm in diameter. The 3D ED data of **TPPM-CPW(Ph)** were collected via precession electron diffraction tomography (PEDT), while a continuous rotation electron diffraction (cRED) data collection protocol was used for **TPPM-CPW(Ph)·BnOH**. Imaging was carried out in STEM mode with a high-angle annular dark field detector (HAADF). The 3D ED data were analyzed using the software PETS2.^49^ The *ab initio* structure determination of **TPPM-CPW(Ph)** and **TPPM-CPW(Ph)·BnOH** was performed with SHELXT.^45^ Data were refined with a fully kinematical approximation, i.e., neglecting dynamical scattering and assuming that *I*_hkl_ is proportional to |*F*_hkl_|^2^. Least-squares structure refinement was performed with the software SHELXL-2014^50^ interfaced with Olex2.^51^

### Powder X-ray Diffraction (PXRD)

The powder X-ray diffraction data used for refinement analysis were collected in a 0.3 mm borosilicate glass capillary, using Cu Kα1 radiation (λ = 1.5406 Å) on a STOE Stadi P equipped with a Ge (111) Johansson focusing monochromator from STOE & Cie and a Mythen2 1K detector from Dectris. Data were preliminarily processed with WinXPOW (STOE & Cie). The Le Bail refinement on powder X-ray diffraction data was conducted with Jana2020.^52^ Manually selected points were used to describe the background, single crystal data (3D ED) were used to define the unit cell, and cyclic refinements on the entire data set were used to generate the profile parameters. The peak profile was modeled as a pseudo-Voigt function, corrected due to axial divergence asymmetry and cut outside the 20*FWHM range. Temperature-resolved *in situ* data collections were performed using a high-temperature attachment for capillaries provided by STOE, collecting each diffraction pattern in a 2θ range of 3.7–22.2° every 10 °C with a heating ramp of 10 °C/min. The PXRD patterns employed in the phase check and comparison analysis were collected using Ni-filtered Cu Kα radiation (λ_Kα1_ = 1.5406 Å, λ_Kα2_ = 1.5444 Å) on a Rigaku SmartLab XE diffractometer equipped with a HyPix-3000 detector. The data were collected in Bragg–Brentano geometry and processed with SmartLab Studio II (Rigaku).

### Thermogravimetric Analyses (TGA)

The thermogravimetric analyses were conducted on a PerkinElmer Instruments model TGA 8000. The experiments were carried out in the temperature range 30–550 °C, with a heating rate of 20 °C min^–1^, and under air flux.

### Gas Sorption Measurements

Gas sorption measurements were performed at −196 °C on a Micromeritics ASAP 2020 surface analyzer. The as-synthesized MOFs were activated under dynamic vacuum (1 × 10^–4^ Pa) at 150 °C for 3 h before the sorption measurements. Langmuir and Brunauer–Emmett–Teller (BET) surface areas were estimated using adsorption points at *p*/*p*_0_ = 0.05–0.15. Pore size distributions were estimated using the MicroActive software by the density functional theory (DFT) method using a slit shape pore model. Gravimetric gas adsorption profiles were obtained using a Mettler Toledo TGA/DSC 3+ using N_2_ as the purge gas and SF_6_ as the measurement gas, and the sample was heated first to 150 °C under N_2_ flow (60 mL min^–1^) for 60 min. The cyclic SF_6_ sorption measurements were subsequently performed at 30 °C with an SF_6_ flow of 60 mL min^–1^ for 20 min. Desorption of SF_6_ for each cycle was carried out under N_2_ flow (60 mL min^–1^) at 100 °C for 20 min.

### Fiber Preparation and Characterization

SPME fibers were prepared according to a procedure previously developed in our laboratories.^53,54^ Briefly, after activation with hydrofluoric acid (40% v/v), 1 cm-long bare fused silica fibers were dipped in the Duralco 4460 epoxy glue and, after 2 min, in 100 mg of **TPPM-CPW(Ph)** ground powder three times. The coating thickness and surface morphology of the SPME fibers were investigated by using scanning electron microscopy (SEM) with a Leica 430i instrument (Leica, Solms, Germany).

### SPME GC-MS Analysis

Fibers were exposed into a canister containing the anesthetics in ambient air for 30 s. Desflurane and sevoflurane were used as model compounds. An HP 6890 Series Plus gas chromatograph (Agilent Technologies, Palo Alto, CA) equipped with an MSD 5973 mass spectrometer (Agilent Technologies) was used for the analyses. Helium was used as the carrier gas at a constant flow rate of 1.2 mL min^–1^; the gas chromatograph was operated in splitless mode with the injector maintained at the temperature of 250 °C. Chromatographic separation was performed on a 30 m × 0.25 mm, df 0.4 μm Rxi-624Sil MS capillary column (Restek, Milan, Italy) using the following temperature program: 40 °C for 15 min and then 15 °C min^–1^ up to 200 °C. The transfer line and source were maintained at temperatures of 220 and 150 °C, respectively. Preliminarily, full scan EI data were acquired to determine appropriate masses for the selected ion monitoring mode (*m*/*z,* 51, 101, and 149 for desflurane; *m*/*z*, 69, 131, and 181 for sevoflurane) under the following conditions: ionization energy: 70 eV; mass range: 35–250 amu; dwell time: 30 ms; and electron multiplier voltage: 1235 V. Signal acquisition and data handling were performed using the HP Chemstation (Agilent Technologies). The commercially available 75 μm carboxen/polydimethylsiloxane (CAR/PDMS) fiber was used to compare the performance of the MOF-based fibers.

## Results and Discussion

### TPPM-CPW(R) Preparation and Characterization

**Figure 2:**
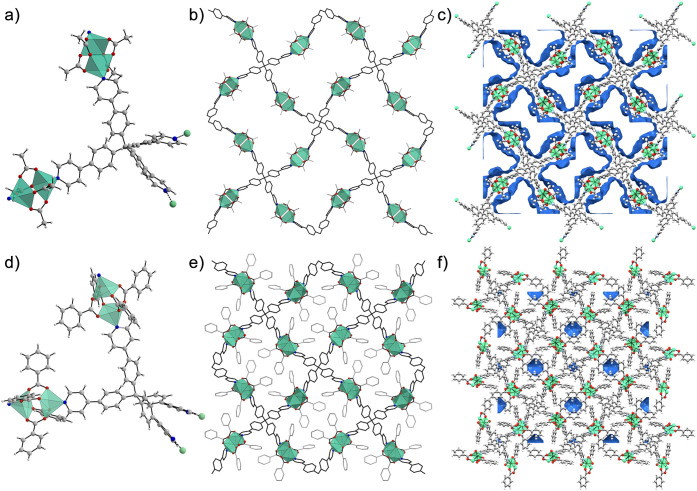
Polymeric repeating unit, crystal structure expansion, and related channel as blue surfaces, respectively, for **TPPM-CPW(Me)** (a–c) and **TPPM-CPW(Ph)** (d–f). The crystal structure expansions are oriented along the crystallographic *b-*axis for **TPPM-CPW(Me)** and the *c-*axis for **TPPM-CPW(Ph)**. Copper atoms are represented as green polyhedra, while oxygen, nitrogen, and carbon atoms are represented as red, blue, and gray spheres, respectively. The solvent molecules were removed for clarity.

To obtain **TPPM-CPW(Me)** as a crystalline phase, two different solutions containing TPPM and [Cu_2_(MeCO_2_)_4_(ACN)_2_], in a solvent mixture of *n*-BuOH/CHCl_3_, were placed in contact through a layering process and heated at 90 °C for 2 days (Scheme S1). The layering approach was essential to obtain a crystalline product. At the end of the reaction, the contact region between the two layers presented large green crystals suitable for SC-XRD analysis (Figure S1). At 200 K, **TPPM-CPW(Me)** exhibits a tetragonal and noncentrosymmetric space group (), with the following unit cell parameters: *a*, *b* = 26.0049(15) Å and *c* = 7.3356(5) Å (Table S1). Its framework is composed of TPPM molecules coordinated through all their pyridyl functionalities by the Cu(II) centers of the SBUs in their apical positions (Figure 2a).

**Figure 3:**
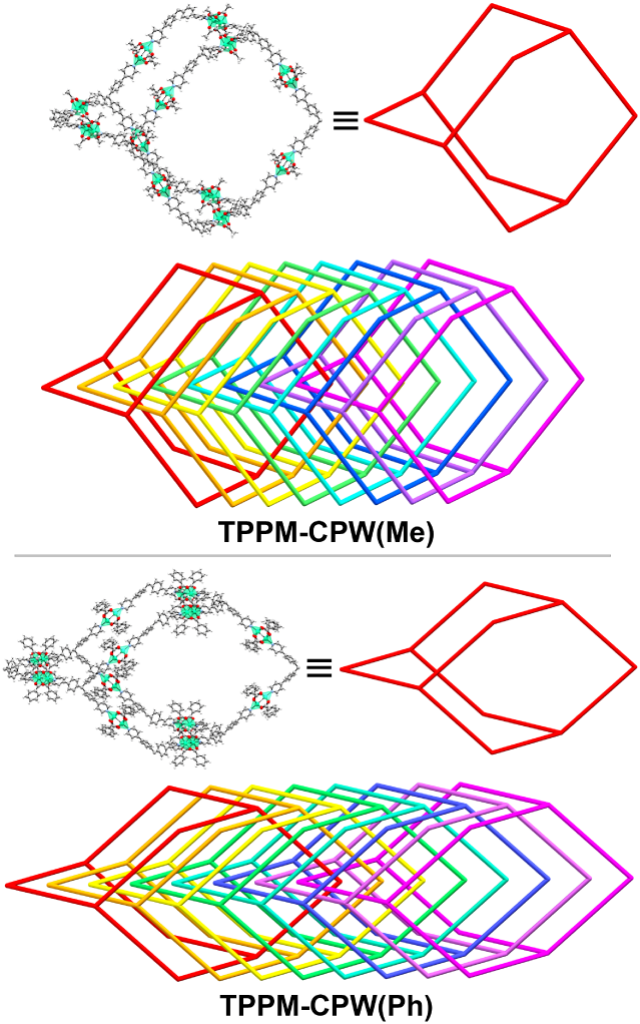
Partial view and schematization of the **dia**-net and its relative 8-fold interpenetration for **TPPM-CPW(Me)** (above) and **TPPM-CPW(Ph)** (below), respectively.

Thus, the obtained MOF presents a 3D polymeric net with {TPPM[Cu_2_(AcCO_2_)_4_]_2_}_*n*_ as a repeating unit. From the X-ray diffraction data, it was also possible to model CHCl_3_ and *n*-BuOH molecules in the pores, located in proximity to the framework backbone (Figures S2 and S3). **TPPM-CPW(Me)** presents a diamond-like network (**dia-**net with point symbol {6^6^}, as calculated by ToposPro software)^55^ eight times interpenetrated (Figure 3). The eight interlaced networks are aligned along the crystallographic *c*-axis with an offset equal to the axis value (Figures 2b and S4). This permits the extension of the channels along that direction (Figure 2c), accounting for ∼45.7% of the unit cell volume (2267.37 Å^3^, as calculated by Mercury4 software).^56^

Different reaction conditions were tested to obtain a **TPPM-CPW(R)** framework featuring [Cu_2_(PhCO_2_)_4_] units and the same connectivity displayed by **TPPM-CPW(Me)** but possessing a phenyl substituent on the carboxylate that could endow the MOF with different properties. A novel crystalline phase was obtained after layering three solutions containing TPPM, benzoic acid, and [Cu_2_(PhCO_2_)_4_(ACN)_2_] in a solvent mixture of ^i^PrOH/CHCl_3_ (1:1 v/v), while heating the system at 50 °C for 3 days (Scheme S1). The layering approach and the use of benzoic acid as a modulating agent proved to be essential for obtaining a crystalline product. The resulting product exhibits rod-shaped micrometer-sized crystals (Figure S9), whose PXRD profile did not correspond to any known structure. Due to the presence of small crystalline domains, 3D electron diffraction (3D ED) was employed for the structural characterization. The analysis was carried out using a special low-dose setup (electron dose below 0.05 el s^–1^ Å^2^). From the reciprocal space reconstruction, it was possible to identify a tetragonal lattice with unit cell parameters *a* = 22.050(6) Å and *c* = 9.0021(3) Å (Figure S10, Table S2). The indexed reflections were then integrated, and the crystal structure was solved *ab initio* in the space group  and kinematically refined, revealing the presence of a 3D coordination polymer based on TPPM and Cu(II) paddle wheels (**TPPM-CPW(Ph)** (Figures 2d and S12), of general formula **{TPPM[Cu**_**2**_**(PhCO**_**2**_**)**_**4**_**]**_**2**_**}**_***n***_. The repeating units form a diamond-like network (**dia-**net) that is eight times interpenetrated as the previously described **TPPM-CPW(Me)**. Moreover, by orienting the **TPPM-CPW(Ph)** along the crystallographic *c*-axis, it is possible to distinguish the phenyl rings of the SBUs protruding toward the center of the pores, in which guest molecules could be entrapped (Figure 2e,f). Unlike **TPPM-CPW(Me)**, this structure does not feature open channels along the *c*-axis. Instead, it displays small and isolated void regions, which constitute 2.9% of the unit cell volume (126.75 Å^3^). No electron density deriving from the presence of the solvent was found, confirming that the structure obtained was the empty form of the MOF (the removal of volatile guests has already been observed under the high vacuum conditions of the TEM column during the 3D ED experiment). Interestingly, the comparison between the **TPPM-CPW(Ph)** PXRD profile calculated from the 3D ED model and the experimental one reveals that they derive from different crystal structures (Figure S20). Moreover, the experimental PXRD diffractogram seems to be correlated to a crystal lattice with a slightly larger unit cell. The empty phase of **TPPM-CPW(Ph)** was also compared to the **TPPM-CPW(Me)** phase at 200 K, revealing similarities in both frameworks. In **TPPM-CPW(Me)** at 200 K, this asymmetry in the distance between the centroids of the SBUs is responsible for a decrease in pore size. Therefore, the empty phase of **TPPM-CPW(Ph)** can be considered as a contracted form of a **TPPM-CPW(R)** MOF constituted of {TPPM[Cu_2_(PhCO_2_)_4_]_2_}_*n*_ repeating units.

### Dynamic Behavior of TPPM-CPW(R)

**Figure 4:**
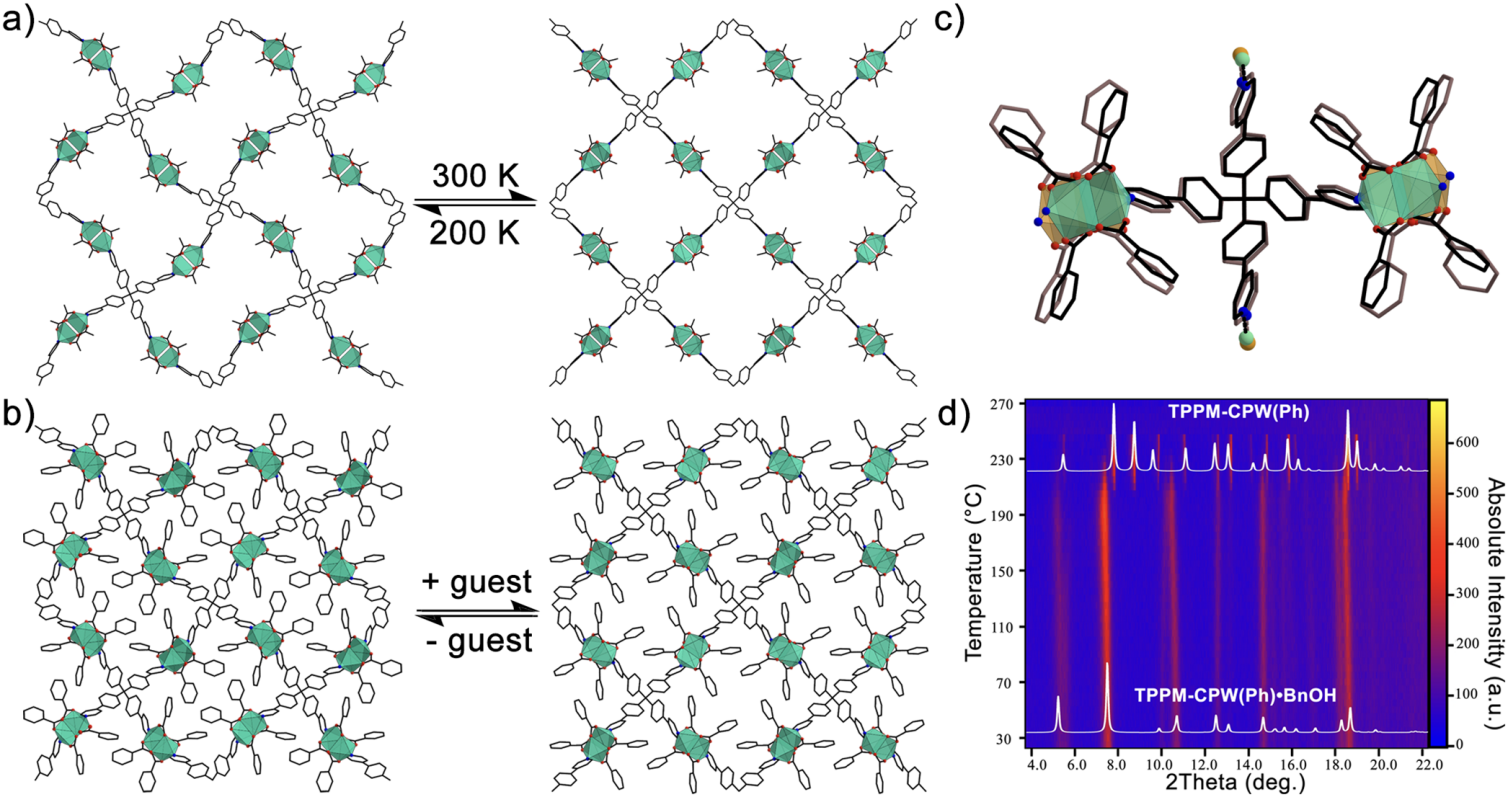
(a, b) Schematization of the phase transition for **TPPM-CPW(Me)** and **TPPM-CPW(Ph)**. (c) Superpositions of the polymeric repeating unit of **TPPM-CPW(Ph)·BnOH** with the **TPPM-CPW(Ph)** empty phase, oriented along the crystallographic *c*-axis. (d) Temperature-induced guest removal of **TPPM-CPW(Ph)·BnOH** analyzed by temperature-resolved *in situ* powder X-ray diffraction, with 2D projection along the intensity axis.

Increasing the temperature to 300 K, **TPPM-CPW(Me)** crystals display different lattice parameters, suggesting the occurrence of a temperature-induced single-crystal-to-single-crystal phase transition (Figure 4a). A complete SC-XRD analysis was carried out at 300 K, highlighting the presence of a tetragonal centrosymmetric space group (*P*4̅/*n*) and *a*, *b* = 27.389(9) Å and *c* = 7.304(2) Å as unit cell parameters (Table S1, Figure S5). This **TPPM-CPW(Me)** structure displays the phenyl rings of the TPPM ligand disordered over two different positions (Figure S6). Moreover, the 300 K form presents larger unit cell values with a volume increase of 518.42 Å^3^ with respect to the 200 K form. This cell expansion also affects the calculated virtual void, which in this case is around 53% of the unit cell volume (2908.93 Å^3^). At 300 K, the system displays square-shaped channels, wherein the SBU centroids’ distances *d*_1_ and *d*_2_ are equal (Figure S7a). The geometry of these channels changes when the temperature is lowered to 200 K. Indeed, the channels undergo a contraction of *d*_1_ and an elongation of *d*_2_, subsequently leading to a reduction in the pore section area (Figure S7b). The distortion of these channels occurs through a rotation of the **TPPM** ligands, causing misalignment between their pyridyl–phenyl wings and the vertical axis of the SBU (Figure S8). The observed contraction of the framework at 200 K highlights the presence of a potentially flexible framework (third-generation MOF). To investigate if the observed temperature-induced phase transition could also occur through solvent removal, crystals of **TPPM-CPW(Me)** were placed under vacuum (∼10^–3^ bar) for 20 min at 300 K. However, the obtained product was completely amorphous. Moreover, when exposed to atmospheric conditions for 2 h, **TPPM-CPW(Me)** drastically loses crystallinity leading to a partially amorphous product (Figure S19). For its susceptibility to the extraction of embedded guest molecules, **TPPM-CPW(Me)** can be categorized as a first-generation MOF.^57^

Framework flexibility upon the removal of guest molecules was also investigated for **TPPM-CPW(Ph)**. To demonstrate that the initial empty phase was the contracted form, it was first necessary to isolate the expanded solvated phase of **TPPM-CPW(Ph)**. To fill its pores with a solvent with a low vapor pressure, the product obtained from the solvothermal reaction was thus soaked in BnOH for a day. This step was intended to prevent the extraction of guest molecules during the 3D ED analysis, which is carried out under the high vacuum conditions of a TEM column (10^–10^ bar).^58^ Interestingly, the same material could also be directly synthesized through a mechanochemical approach. The reaction was conducted in a mixer mill operating at 20 Hz, for 30 min, using BnOH as a liquid additive (Scheme S1). This environmentally friendly approach is also very effective in terms of efficiency, allowing us to achieve large quantities of **TPPM-CPW(Ph)·BnOH** in a few minutes. However, the mechanochemical product shows broader diffraction peaks attributable to smaller crystalline domains than the solvothermal product (Figure S21). For this reason, 3D ED analysis was carried out on the product obtained from the soaking process.

The 3D ED data collection was conducted on a single nanocrystal (Figure S9) through a STEM-cRED data collection protocol. The reconstructed reciprocal space exhibited a different symmetry with respect to the contracted, empty phase of **TPPM-CPW(Ph)**. The collected data were indexed with a tetragonal unit cell featuring the cell parameters *a =* 23.049(2) Å and *c* = 8.8059(11) Å (Figure S11). The structure could be solved *ab initio* in the *P*4/*n* space group and refined with a kinematical approach. The resulting structure corresponds to a MOF with the same connectivity as the empty, contracted form of **TPPM-CPW(Ph)** (Figures 4a,b and S13, Table S3). However, compared to the empty phase, the {TPPM[Cu_2_(PhCO_2_)_4_]_2_}_*n*_ repeating units show differences in their conformational arrangement. Indeed, in this new phase, the pyridyl–phenyl wings are slightly distorted, leading to a shift in the position of the metal nodes (Figure 4b,c). Along the *a* and *b* axes, the centroids of the Cu(II) paddle wheels are now equally distanced, resulting in a square-like geometry that is comparable to that of **TPPM-CPW(Me)** at 300 K (Figure S14). This positional change in the SBU induces a rearrangement of their phenyl substituents, leading to the opening of the pores that run along the *c*-axis (13.1% of the unit cell volume, 614.5 Å^3^, Figure S16). Thus, **TPPM-CPW(Ph)·BnOH** can be considered as the expanded phase of **TPPM-CPW(Ph)**.

The Fourier difference map calculation over the kinematically refined **TPPM-CPW(Ph)·BnOH** structure obtained from 3D ED analysis allowed the identification of a broad region in the channels in which the solvent molecules can be located (Figure S15). Moreover, a TGA analysis conducted on this material showed the removal of the solvent molecules before thermal degradation (Figure S24). The solvent stoichiometry (SS^TGA^) calculated from its weight loss resulted in around six molecules of BnOH per repeating unit. However, this value of SS^TGA^ is too high to solely correspond to the removal of solvent molecules trapped in the MOF channels. Indeed, from the first derivative of the TGA thermogram, we can clearly distinguish the presence of two different processes at 90 and 114 °C. The excess of BnOH and the presence of two thermal desorption processes might be correlated to a fraction of solvent trapped on the surface of the **TPPM-CPW(Ph)·BnOH** nanocrystals. The calculated PXRD profile of the **TPPM-CPW(Ph)·BnOH** phase agreed with the experimental pattern, as further confirmed by Le Bail refinement conducted from the lattice parameters obtained through the 3D ED analysis (Figure S22). The resulting unit cell values were *a*, *b* = 23.2663(8) Å and *c* = 8.8096(5) Å, indicating the congruence of the two crystallographic characterizations (Table S4). Moreover, temperature-resolved *in situ* PXRD analysis was conducted to investigate the presence of a phase transition between **TPPM-CPW(Ph)·BnOH** and the **TPPM-CPW(Ph)** empty phase. From the collected diffractograms, the presence of a phase transition around 210 °C is evident, which involves the transformation of **TPPM-CPW(Ph)·BnOH** in **TPPM-CPW(Ph)**, followed by the amorphization of the sample at 250 °C (Figure 4d). The discrepancies in temperature between TGA and the temperature-resolved *in situ* PXRD analysis might be correlated to the different conditions under which they have been carried out. This transformation is associated with a temperature-induced desolvation process, moving from an expanded, filled phase (**TPPM-CPW(Ph)·BnOH**) to the empty contracted **TPPM-CPW(Ph)** one. The loss of benzyl alcohol has been demonstrated by both TGA analysis (Figure S25) and NMR spectroscopy on the digested MOF (Figure S26). The observed phase transition denotes the presence of a flexible framework capable of rearranging its nets after the desolvation process. To compensate the reduction of interactions after the guest extraction, the material undergoes an overall conformational rearrangement of its TPPM pyridyl–phenyl arms, followed by an increase in interframework distances between the interlaced nets (Figures S17, S18). Remarkably, the contraction of the framework upon guest removal allows the classification of **TPPM-CPW(Ph)** as a third-generation MOF.^57^

### Gas Sorption Measurements for TPPM-CPW(Ph)

**Figure 5:**
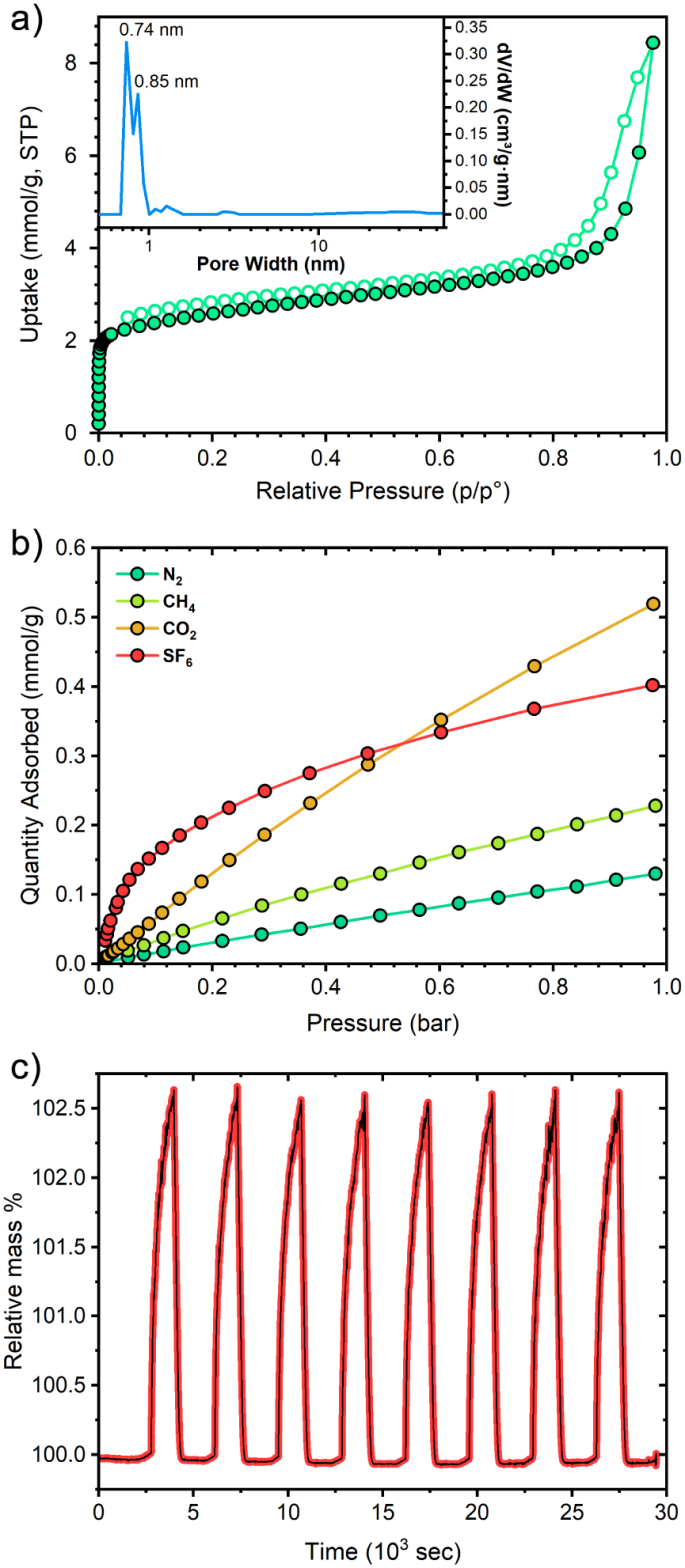
(a) Nitrogen adsorption isotherms of **TPPM-CPW(Ph)** and its relative pore size distribution. The measurement was conducted at liquid N_2_ temperature. (b) N_2_, CH_4_, CO_2_, and SF_6_ gas adsorption isotherms of **TPPM-CPW(Ph)** measured at 293 K. (c) SF_6_ adsorption/desorption cycles on **TPPM-CPW(Ph)**.

Gas sorption measurements were conducted on the **TPPM-CPW(Ph)** contracted phase obtained from mechanochemical synthesis. The nitrogen adsorption isotherm at 77 K (Figure 5a) shows an initial Langmuir-type adsorption region, which reflects the presence of micropores, while the subsequent increase of the amount of nitrogen adsorbed can be ascribed to the presence of mesopores followed by a pronounced interparticle condensation. The experimental Langmuir and BET surface area values were found to be 260 and 206 m^2^ g^–1^, respectively (Table S5). The analysis revealed a pore volume value of 0.29 cm^3^ g^–1^, and from the pore size distribution plot, it is possible to distinguish the presence of a bimodal distribution, related to pores with diameters of 0.74 and 0.85 nm (Figure 5a, inset). The nitrogen adsorption at low relative pressures displayed in the micropore region is compatible with the expansion of the initial **TPPM-CPW(Ph)** contracted, empty phase. These results confirm again that the **TPPM-CPW(Ph)** empty phase can be converted to the filled phase when exposed to a guest, nitrogen in this case, underlining the reversibility of the conversion process between its empty and contracted forms.

**TPPM-CPW(Ph)** was further tested for the gas sorption of greenhouse gases such as CO_2_, CH_4_, and SF_6_. Single-component isotherms were collected at 293 K and up to 1 bar for these three gases, and nitrogen was also tested for comparison (Figure 5b). Sulfur hexafluoride (SF_6_), a synthetic inert gas extensively utilized in power and semiconductor industries for its superior dielectric properties, arc-quenching capabilities, thermoacoustic insulation, and efficacy as a plasma etchant, represents a significant environmental concern as its global warming potential is 23 900 times that of CO_2_ and it has an estimated atmospheric lifetime of approximately 3200 years.^59^ Microporous frameworks have been extensively investigated as selective materials for SF_6_ capture and separation, as their pores can be engineered to enhance SF_6_ adsorption performance.^60^ Among the proposed adsorbents, MOFs with 5.5–8.5 Å micropores decorated with benzene rings were found to accommodate SF_6_ (5.2 Å), thanks to synergistic interactions, namely, (i) multiple van der Waals interactions between the F atoms of SF_6_ molecules and C–H groups of the aromatic rings and (ii) F−π interactions between F atoms and the large electron cloud of the delocalized π-system.^61,62^ The channels of the expanded form of **TPPM-CPW(Ph)** nicely match these characteristics, as highlighted by the potential electrostatic surface reported in Figure S29. Indeed, despite the modest surface area, **TPPM-CPW(Ph)** presents a pronounced adsorption of SF_6_ at low pressure, suggesting a moderate affinity of the framework toward this gas. Moreover, this material displays a completely reversible uptake and release of SF_6_ for up to 8 cycles (Figure 5c). The unusual SF_6_ adsorption properties of **TPPM-CPW(Ph)** prompted us to investigate the affinity of this framework for other fluorinated guests, such as fluorinated ethers used as anesthetics in clinical practice.

### SPME-GC-MS Analysis on Fluorinated Anesthetics with TPPM-CPW(Ph)

**Figure 6:**
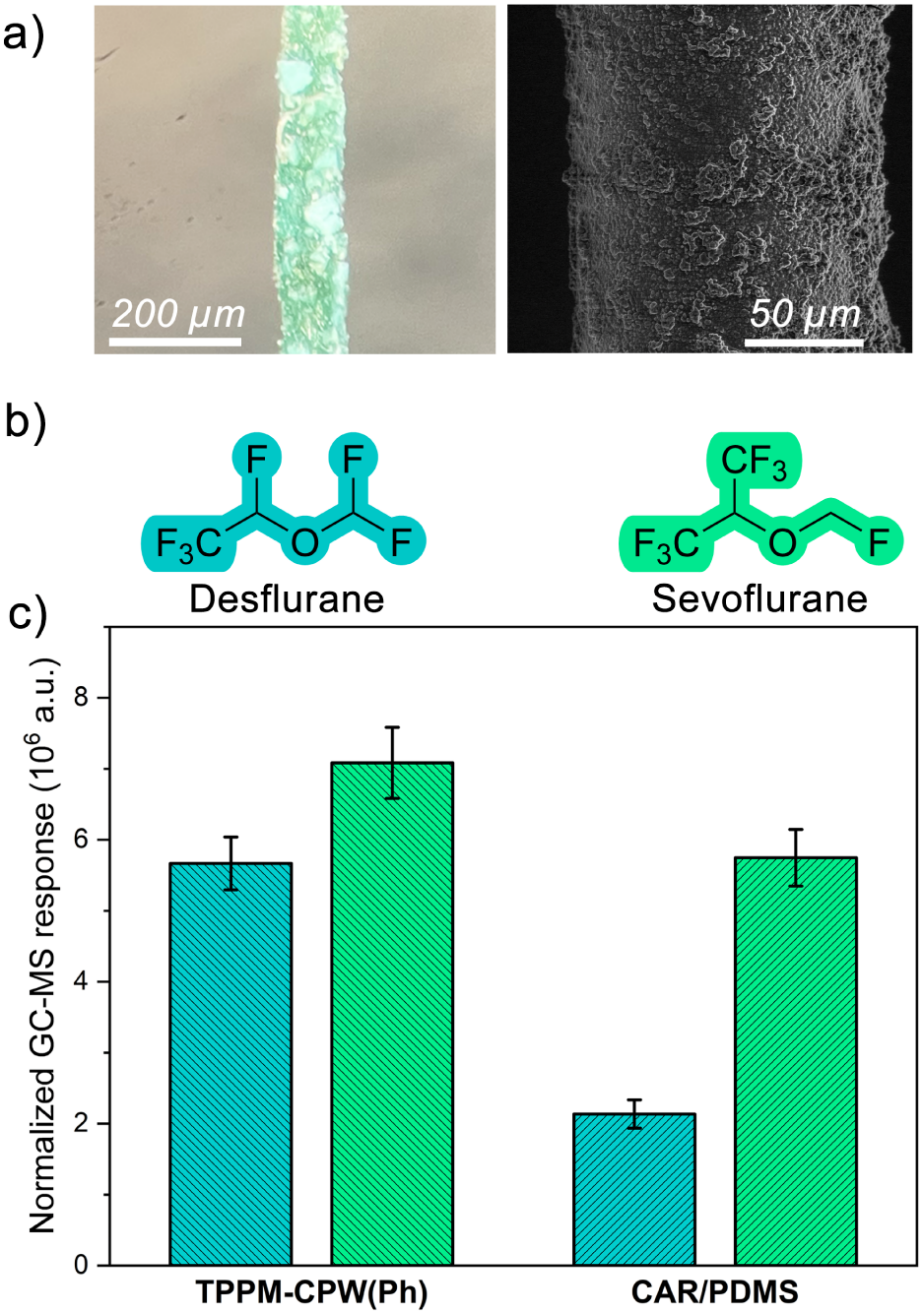
(a) Optical microscope (left) and SEM (right) images of SPME fiber functionalized with **TPPM-CPW(Ph)**. (b) Molecular sketch of desflurane and sevoflurane. (c) Normalized GC-MS response: **TPPM-CPW(Ph)**-based fiber vs 75 μm CAR/PDMS fiber.

MOFs have been extensively applied to air purification and monitoring,^63,64^ given their remarkable surface areas and tunable pore sizes, which favor the adsorption and diffusion of analyte molecules. In particular, several MOF-based sensors for air pollutant detection based on optical, electrochemical, capacitive, and gravimetrical signal transduction have been reported so far.^65^ Conversely, the MOF adsorption of volatile fluorocarbons such as sevoflurane and desflurane (Figure 6b), which are volatile anesthetics (VAs) used in modern surgery, is still unexplored. Although hospital ventilation systems and scavenging devices limit indoor exposure to waste gases, they still release VAs into the atmosphere.^66^ These gases, especially desflurane, are potent greenhouse gases (GHGs), with their atmospheric concentrations increasing significantly over the past decade. In 2014, desflurane accounted for about 80% of VA emissions, contributing to approximately 3 million tons of CO_2_ equivalents, positioning hospitals as notable GHG emission sources.^67^ MOFs have been proposed as adsorbents for VAs,^68^ in particular for the recovery of anesthetic xenon,^69−71^ but their application to the detection of fluorinated VAs in air is still unexplored. As a proof-of-concept, **TPPM-CPW(Ph)** was used as an SPME coating for the extraction of desflurane and sevoflurane in ambient air. A schematic representation of the procedure utilized in this study is reported in Figure S30. The fibers were investigated by means of SEM, demonstrating the development of thin (16 ± 3 μm thickness) and uniform coatings (Figure 6a). TGA analysis demonstrated the good thermal stability of the material, from room temperature to 300 °C under air flux, with a weight loss of about 26% due to solvent removal (Figure S24), thus allowing its use for thermal desorption of the analytes. Finally, no significant bleeding was observed by desorbing the fiber into the GC injection port at 250 °C under a He atmosphere. In addition, PXRD analyses conducted on the thermally treated fiber highlight that **TPPM-CPW(Ph)** completely retains its crystallinity after this process (Figure S23).

The optimal condition for the extraction of the investigated anesthetics was found to correspond to an extraction time of 30 s (Figure S31). In fact, a decrease in the GC responses was observed with extraction times higher than 30 s, probably due to the high vapor pressure of the investigated analytes, which are partially desorbed from the MOF. A good repeatability was also observed, obtaining CV always lower than 3% (*n* = 10). Preliminary extraction experiments carried out using a fiber coating only made by the epoxy glue demonstrated that both sevoflurane and desflurane were not extracted. Therefore, the **TPPM-CPW(Ph)** material proved to be adequate for the extraction of the considered anesthetics.

Finally, the performance of the developed coating toward the extraction of sevoflurane and desflurane was compared with that of the commercially available CAR/PDMS 75 μm fiber. As shown in Figure 6c, despite the reduced coating thickness, the **TPPM-CPW(Ph)**-based fiber was characterized by the highest extraction capability, thus suggesting its potential use for the evaluation of occupational exposure in the operating rooms.

## Conclusions

In this study, two isoreticular flexible MOFs, **TPPM-CPW(Me)** and **TPPM-CPW(Ph)**, were synthesized by combining the tetrahedral ligand TTPM with linear Cu(II)-based SBUs, demonstrating reversible phase transitions between open and closed pore states under external stimuli such as temperature and vacuum. The stability of these MOFs upon the removal of guest molecules was significantly influenced by the nature of the residual groups attached to the SBU, with **TPPM-CPW(Ph)** behaving as a third-generation soft porous crystal. The presence of phenyl rings protruding into the pores significantly enhanced **TPPM-CPW(Ph)** stability and adsorption affinity toward fluorinated guests such as gaseous SF_6_ and volatile anesthetics such as desflurane and sevoflurane. Furthermore, this material was tested as a SPME fiber coating, showing superior preconcentration capability for these fluorinated VAs in air compared to commercially available CAR/PDMS fibers. Overall, this work highlights the versatility of this class of flexible MOFs in terms of framework functionalization and property tunability, emphasizing their potential application in the capture of volatile fluorinated compounds.

## Supporting Information Available

Crystallographic data (CIF)
Synthetic procedures for [Cu_2_(CH_3_CO_2_)_4_(ACN)_2_] and [Cu_2_(PhCO_2_)_4_(ACN)_2_] preparation, **TPPM-CPW(R)** MOF structural characterization data (SC-XRD, 3D ED and TEM analysis, PXRD), TGA graphs, NMR spectrum of digested**TPPM-CPW(Ph)**, gas sorption data, QM calculations and potential electrostatic surfaces, SPME-GC-MS analysis data for the evaluation of the extraction time (PDF)

## Supplementary Material

cm4c03221_si_001.cif

## Accession Codes

The crystallographic data have been deposited with the Cambridge Crystallographic Data Centre as supplementary publication no. 2404704–2404707 and can be obtained free of charge on application to the CCDC, 12 Union Road, Cambridge, CB2 1EZ, UK (e-mail: deposit@ccdc.cam.ac.uk or http://www.ccdc.cam.ac.uk).
