# Supplementary material for: A Flexible Interpenetrated Diamondoid Metal–Organic Framework with Aromatic-Enriched Channels as a Preconcentrator for the Detection of Fluorinated Anesthetics
Source: Chem Mater. 2025 Mar 11;37(6):2230–40. doi: 10.1021/acs.chemmater.4c03221 (PMC11949194; doi:10.1021/acs.chemmater.4c03221)
Supplement: Supplementary file 2 — cm4c03221_si_002.pdf [file cm4c03221_si_002.pdf]

Supporting Information

A Flexible Interpenetrated Diamondoid Metal-Organic Framework with Aromatic-Enriched Channels as a Preconcentrator for the Detection of Fluorinated Anesthetics

Danilo Marchetti,[a,d] Nicolò Riboni,[a] A. Ken Inge,[b] Ocean Cheung,[c] Mauro Gemmi,[d] Enrico Dalcanale,[a] Federica Bianchi,[a] Chiara Massera,[a,*] Alessandro Pedrini[a,*]

[a]Department of Chemistry, Life Sciences and Environmental Sustainability, INSTM UdR Parma, University of Parma, Parco Area delle Scienze 17/A, Parma 43124, Italy

[b]Department of Materials and Environmental Chemistry, Stockholm University, Frescativägen 8, Stockholm 10691, Sweden

[c]Division of Nanotechnology and Functional Materials, Department of Materials Science and Engineering, Ångström Laboratory, Uppsala University, Lägerhyddsvägen 1, Uppsala 75103, Sweden

[d]Center for Materials Interfaces, Electron Crystallography, Istituto Italiano di Tecnologia, Viale Rinaldo Piaggio 34, Pontedera 56025, Italy

*Email: chiara.massera@unipr.it, alessandro.pedrini@unipr.it

Table of contents

1 Synthetic Procedures *S2*

1.1 [$Cu_2(CH_3CO_2)_4(ACN)_2$] Synthesis *S2*

1.2 [$Cu_2(PhCO_2)_4(ACN)_2$] Synthesis *S2*

2 Structural Characterization *S4*

2.1 Single Crystal X-ray Diffraction (SC-XRD) *S4*

2.2 3D Electron Diffraction (3D ED) and TEM analysis *S8*

2.3 Powder X-ray Diffraction (PXRD) *S14*

3 Thermogravimetric Analyses (TGA) *S17*

4 NMR Characterization *S18*

5 Gas sorption measurements *S19*

6 QM Calculations *S20*

7 SPME-GC-MS analysis of fluorinated anaesthetics *S21*

1 Synthetic Procedures

1.1 [$Cu_2(CH_3CO_2)_4(ACN)_2$] Synthesis

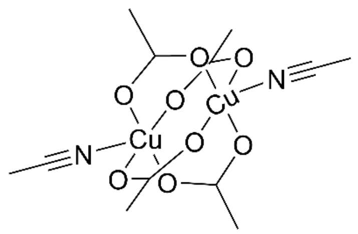

[$Cu_2(CH_3CO_2)_4(ACN)_2$]

The synthesis was conducted solubilizing copper acetate monohydrate (500 mg) in 200 mL of ACN. Molecular sieves (3 Å) were added to the solution. After 2h, the solution was filtered, and the solvent evaporated under reduced pressure. The product was obtained as large dark-green crystals suitable for single crystal X-ray diffraction. The solid-state analysis confirmed the structure of the paddle-wheel of general formula **[$Cu_2(CH_3CO_2)_4(ACN)_2$]·ACN** (quantitative yield, CSD ref. code: HILNUL).

1.2 [$Cu_2(PhCO_2)_4(ACN)_2$] Synthesis

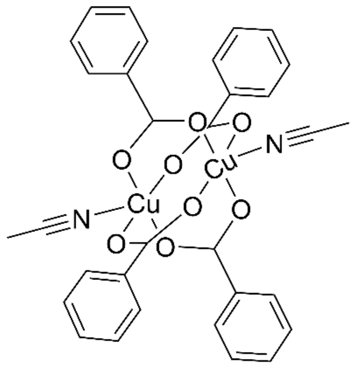

[$Cu_2(PhCO_2)_4(ACN)_2$]

The synthesis was conducted by mixing into an agate mortar benzoic acid (500 mg, 4.1 mmol) and sodium hydroxide (164 mg, 4.1 mmol). Then, 500 µL of water were added and a liquid assisted grinding (LAG) was performed for 10 minutes. In the meantime, copper sulphate pentahydrate (511 mg, 2.1 mmol) was added to 5 mL of water and sonicated for 20 minutes until complete solubilization. The mechanochemical product was then solubilized in 10 mL of water and the copper sulphate solution was added, immediately obtaining a blue precipitate. The mixture was cooled into an ice bath and the precipitated was filtered. The obtained solid was subsequently solubilized in 200 mL of ACN and molecular sieves (3 Å) were added to the mixture. After 2h, the reaction mixture was filtered, and the solvent evaporated under reduced pressure. The product was then recrystalized at 4°C, isolating

blue crystals suitable for single crystal X-ray diffraction. The solid-state analysis confirmed the structure of the paddle-wheel of general formula **[$Cu_2(PhCO_2)_4(ACN)_2$]•ACN** (376 mg, 51% yield, CSD ref. code: QILXOY).

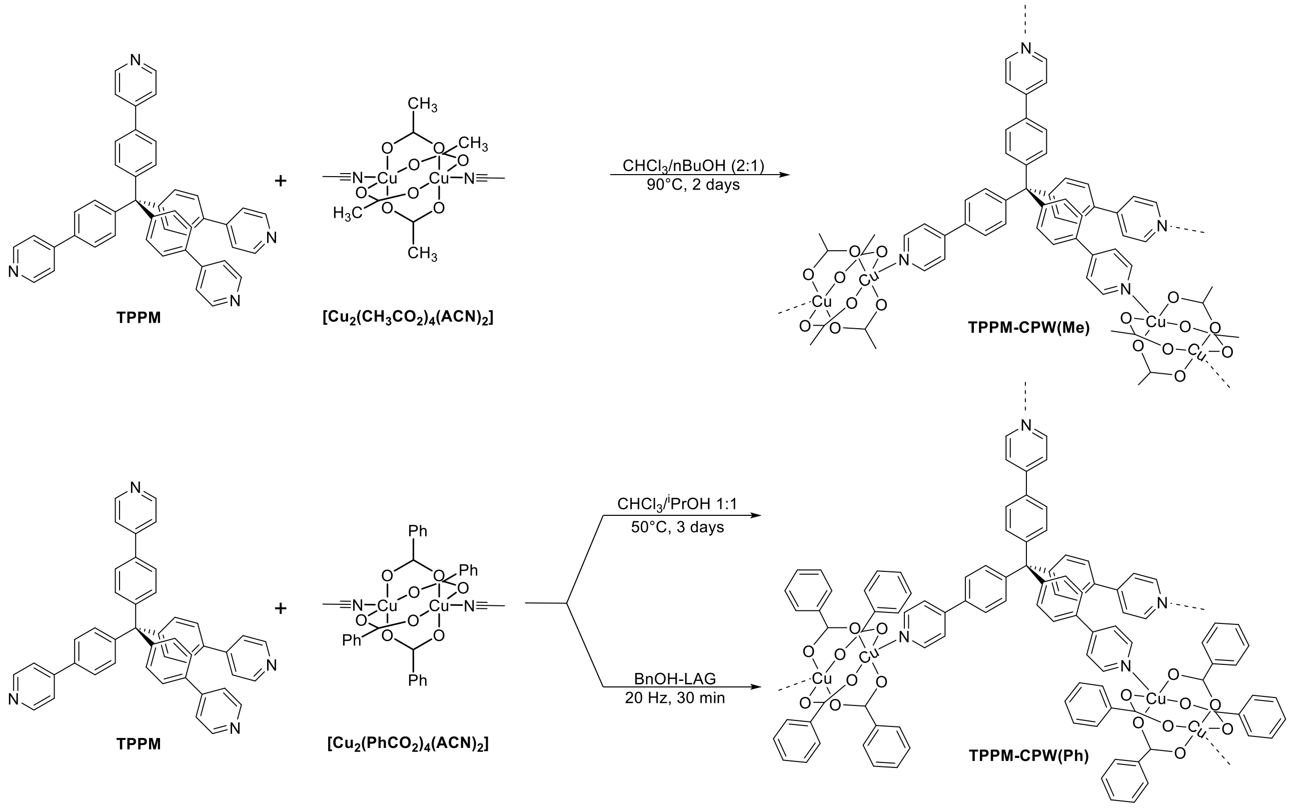

Scheme S1. TPPM-CPW(R) MOFs reaction scheme: R = CH_3 (*above*), R = Ph (*below*).

2 Structural Characterization

2.1 Single Crystal X-ray Diffraction (SC-XRD)

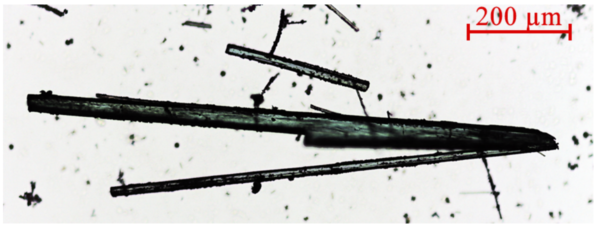

Figure S1. Optical Microscope image of **TPPM-CPW(Me)** crystals.

Table S1. Crystallographic information for **TPPM-CPW(Me)** at 200 and 300 K.

Empirical formula	$C_{61}H_{56}N_4O_{16}Cu_4 \cdot CHCl_3 \cdot C_4H_9OH$	$C_{61}H_{56}N_4O_{16}Cu_4$
Formula weight	1548.74	1355.25
Temperature/K	200(2)	300(2)
Crystal system	tetragonal	tetragonal
Space group	P-4	P4/n
a/Å	26.0049(15)	27.389(9)
b/Å	26.0049(15)	27.389(9)
c/Å	7.3356(5)	7.304(2)
α/°	90	90
β/°	90	90
γ/°	90	90
Volume/Å^3	4960.7(7)	5479(4)
Z	2	2
ρ_{calc}g/cm^3	1.037	0.821
μ/mm^{-1}	2.114	1.203
F(000)	1588	1388.0
Radiation	CuKα (λ = 1.54178)	CuKα (λ = 1.54178)
2θ range for data collection/°	4.806 to 118.23	4.562 to 109.9
Index ranges	$-28 \le h \le 28, -28 \le k \le 28, -6 \le l \le 8$	$-28 \le h \le 28, -25 \le k \le 28, -7 \le l \le 6$
Reflections collected	25467	19208
Independent reflections (R_{int})	7110 (0.1146)	3392 (0.1478)
Observed reflections	5365	1488
Data/restraints/parameters	7110/0/469	3392/3/230
Goodness-of-fit on F^{2a}	1.010	1.109
$R_{indices}$ [F_o>4σ(F_o)]^bR_1, wR_2	R_1 = 0.0863, wR_2 = 0.2147	R_1 = 0.1185, wR_2 = 0.3510
Largest diff. peak/hole / e Å^{-3}	0.616, -0.738	0.409/-0.543

[a]Goodness-of-fit S = $[\Sigma w(F_o^2-F_c^2)^2/ (n-p)]1/2$, where n is the number of reflections and p the number of parameters.

[b]$R_1 = \Sigma||F_o|-|F_c||/\Sigma|F_o|$, $wR_2 = [\Sigma[w(F_o^2-F_c^2)^2]/\Sigma[w(F_o^2)^2]]^{1/2}$.

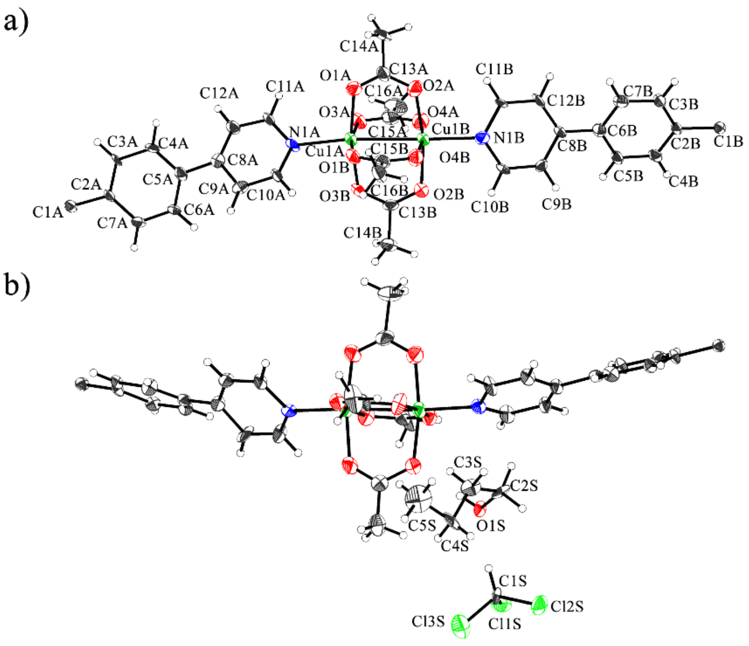

Figure S2. Ortep view of the asymmetric unit of **TPPM-CPW(Me)** at 200 K (probability level 50%). To highlight all the atoms involved, the asymmetric unit is represented in two different orientations. In (a), solvent molecules have been removed for clarity.

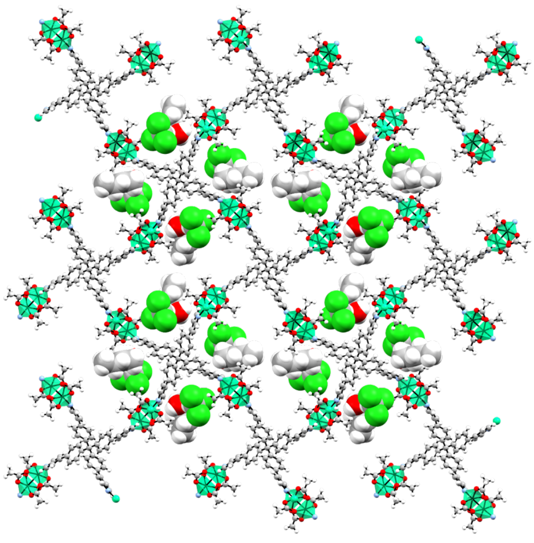

Figure S3. Packing of the **TPPM-CPW(Me)** crystal structure oriented along the crystallographic *c*-axis. Copper atoms are represented as green ellipsoids, while the solvent molecules embedded in the channel are depicted as CPK models.

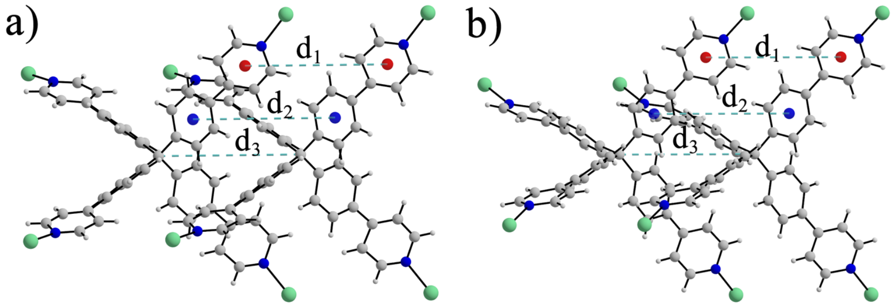

Figure S4. View of the principal inter-framework distances along the *c*-axis for the two TPPM fragments in the asymmetric unit of **TPPM-CPW(Me)** at 200 K: fragment named A (a) and fragment named B (b), see Figure S2. Pyridyl rings and phenyl rings centroids are represented in red and blue, respectively. The distances $\mathbf{d_1}$ (pyridyl centroids distances), $\mathbf{d_2}$ (phenyl centroid distances) and $\mathbf{d_3}$ (C1···C1 distances) are equal for both fragments and equivalent to 7.3356(5) Å.

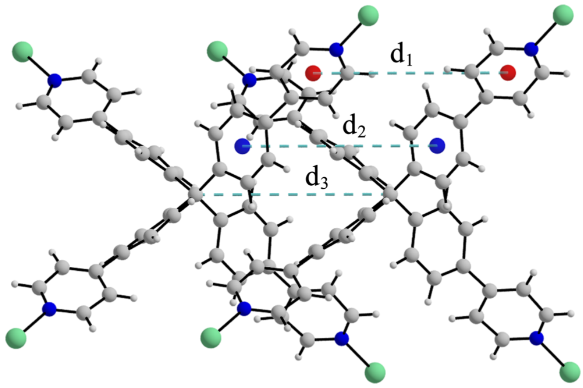

Figure S5. View of the principal inter-framework distances along the *c*-axis of **TPPM-CPW(Me)** at 300K. Pyridyl rings and phenyl rings centroids are represented in red and blue, respectively. The distances $\mathbf{d_1}$ (pyridyl centroids distances), $\mathbf{d_2}$ (phenyl centroid distances) and $\mathbf{d_3}$ (C1··C1 distances) are equal for both fragments and equivalent to 7.304(2) Å.

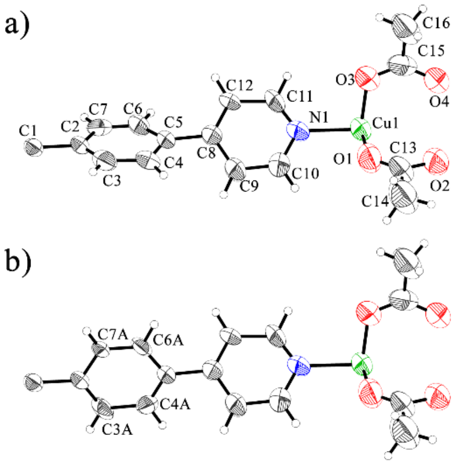

Figure S6. Ortep view of the asymmetric unit of **TPPM-CPW(Me)** at 300K (probability level 30%). Two different views of the structure are reported, to highlight the presence of positional disorder on the **TPPM** phenyl ring.

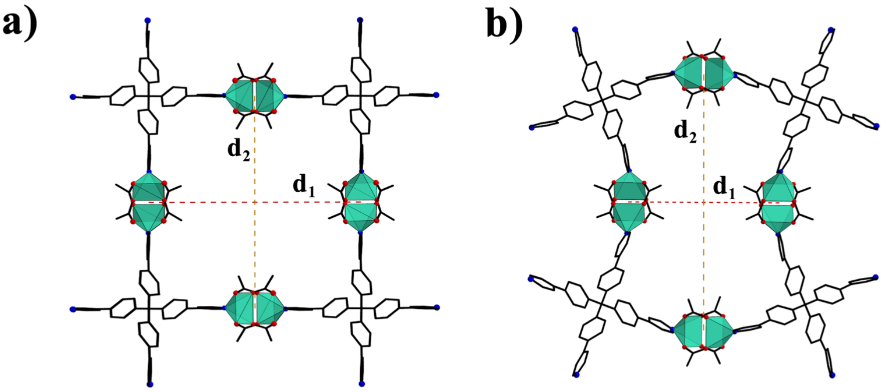

Figure S7. Comparison of the two pore geometries in **TPPM-CPW(Me)** at 300 K (a) and 200 K (b), both oriented along the *c*-axis. The distances between the SBU centroids are depicted as red ($\mathbf{d_1}$) and orange ($\mathbf{d_2}$) dashed lines. $\mathbf{d_1}$(a) = $\mathbf{d_2}$(a) = 19.367(5) Å, $\mathbf{d_1}$(b) = 13.455(2) Å; $\mathbf{d_2}$(b) = 23.225(1) Å. Hydrogen atoms, disordered fragments and solvent molecules have been omitted for clarity.

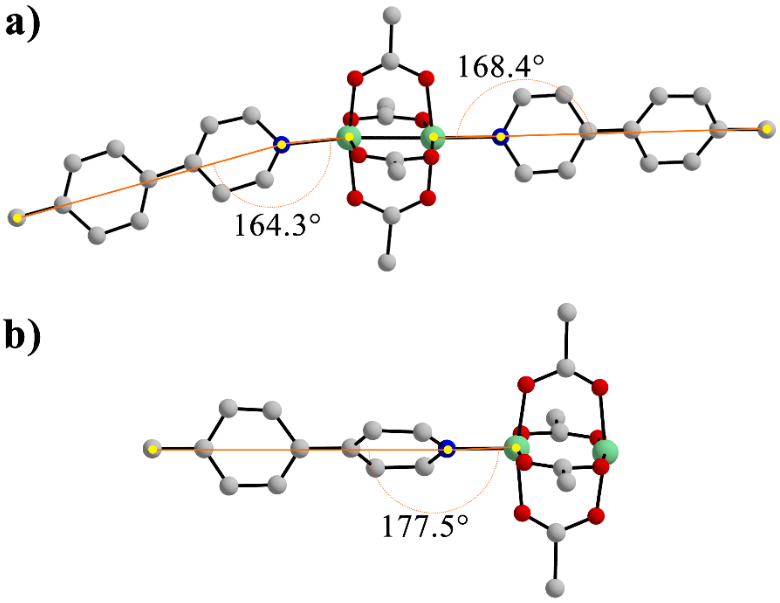

Figure S8. Asymmetric unit of **TPPM-CPW(Me)** at 200 K (a) and 300 K (b). The angle C1-N1-Cu1 highlights the differences in terms of coordination geometry between the two structures.

2.2 3D Electron Diffraction (3D ED) and TEM analysis

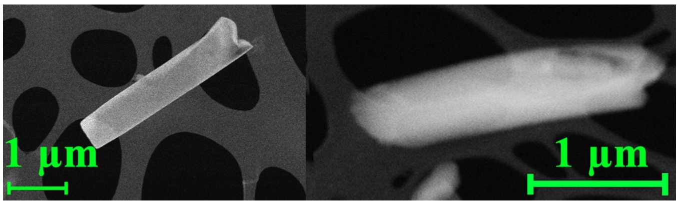

Figure S9. HAADF-STEM image of the **TPPM-CPW(Ph)** nanocrystals used for the 3D ED data collection. (*right*) **TPPM-CPW(Ph)** empty phase, (*left*) **TPPM-CPW(Ph)•BnOH** phase.

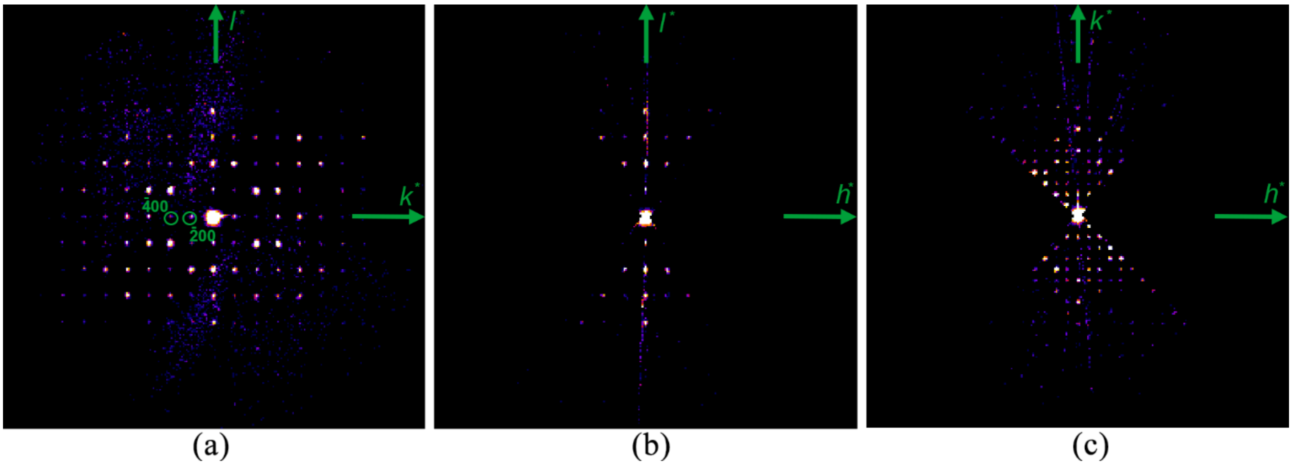

Figure S10. Reciprocal space sections of the **TPPM-CPW(Ph)** 3D ED data collection, reconstructed with PETS2 from the 3D ED data: (a) *0kl*, (b) *h0l*, (c) *hk0*. The *0kl* reciprocal plane section shows the extinction rule $k = 2n$

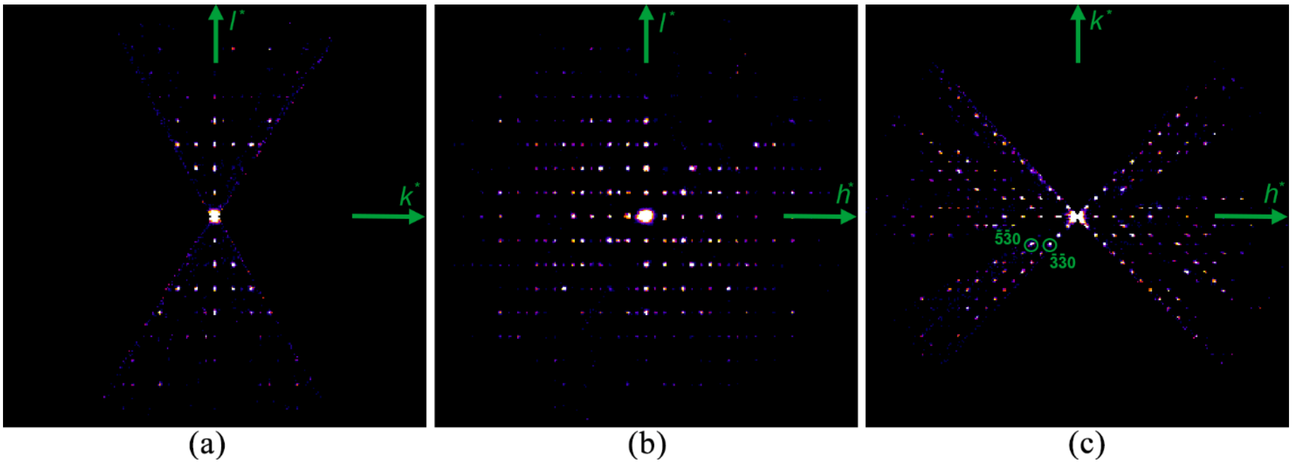

Figure S11. Reciprocal space sections of the **TPPM-CPW(Ph)•BnOH** 3D ED data collection, reconstructed with PETS2 from the 3D ED data: (a) *0kl*, (b) *h0l*, (c) *hk0*. The *hk0* reciprocal plane section shows the extinction rule $h + k = 2n$.

Table S2. Crystallographic information for **TPPM-CPW(Ph)**

Empirical formula	$C_{101}H_{72}Cu_4N_4O_{16}$
Formula weight	462.97
Temperature/K	293(2)
Crystal system	tetragonal
Space group	*P*-4*b*2
a/Å	22.050(6)
b/Å	22.050(6)
c/Å	9.0021(3)
α/°	90
β/°	90
γ/°	90
Volume/Å^3	4386(3)
Z	2
ρ_{calc}g/cm^3	1.402
F(000)	709
Radiation	electrons (λ = 0.0335 Å)
2θ range for data collection/°	0.174 to 1.918
Index ranges	$-16 \leq h \leq 17$, $-21 \leq k \leq 18$, $-8 \leq l \leq 8$
Reflections collected	8108
Independent reflections	2274 [R_{int} = 0.3549, R_{sigma} = 0.2945]
Data/restraints/parameters	2274/59/127
Goodness-of-fit on F^2	1.129
Final R indexes [I>=2σ (I)] [a]	R_1 = 0.1855, wR_2 = 0.4373
Final R indexes [all data] [a]	R_1 = 0.3005, wR_2 = 0.5137

[a] $R_1 = \Sigma \| F_o | - | F_c \| / \Sigma | F_o |$, $wR_2 = [\Sigma[w(F_o^2-F_c^2)^2]/\Sigma[w(F_o^2)^2]]^{1/2}$

Table S3. Crystallographic information for **TPPM-CPW(Ph)•BnOH**

Empirical formula	$C_{101}H_{72}Cu_4N_4O_{16}$
Formula weight	1851.88
Temperature/K	298(2)
Crystal system	tetragonal
Space group	*P*4/n
a/Å	23.049(2)
b/Å	23.049(2)
c/Å	8.8059(10)
α/°	90
β/°	90
γ/°	90
Volume/Å^3	4678.3(11)
Z	2
ρ_{calc}g/cm^3	1.3015
F(000)	709.0
Radiation	electrons (λ = 0.0335 Å)
2θ range for data collection/°	0.218 to 2.302
Index ranges	$-27 \leq h \leq 26$, $-21 \leq k \leq 21$, $-10 \leq l \leq 10$
Reflections collected	12540
Independent reflections	3733 [R_{int} = 0.2925, R_{sigma} = 0.2492]
Data/restraints/parameters	3733/64/127
Goodness-of-fit on F^2	1.043
Final R indexes [I>=2σ (I)] [a]	R_1 = 0.2215, wR_2 = 0.5671
Final R indexes [all data] [a]	R_1 = 0.2966, wR_2 = 0.6469

[a] $R_1 = \Sigma||F_o| - |F_c||/\Sigma|F_o|$, $wR_2 = [\Sigma[w(F_o^2-F_c^2)^2]/\Sigma[w(F_o^2)^2]]^{1/2}$

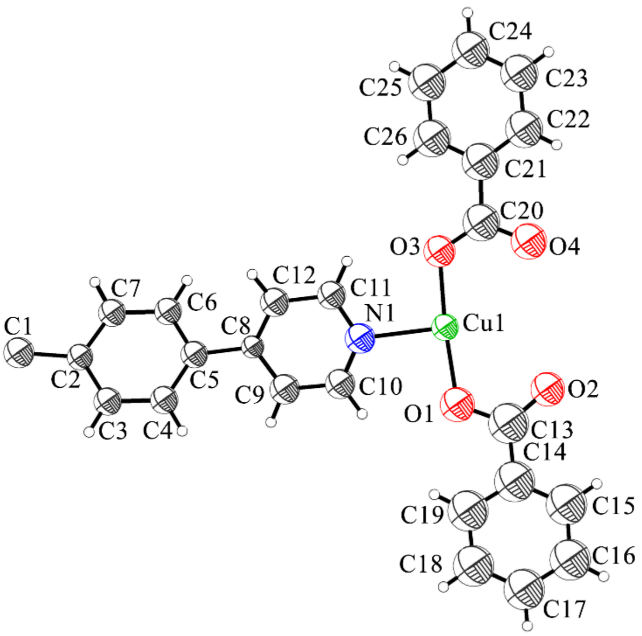

Figure S12. Ortep view of the asymmetric unit of **TPPM-CPW(Ph)** empty phase. Probability level 30%.

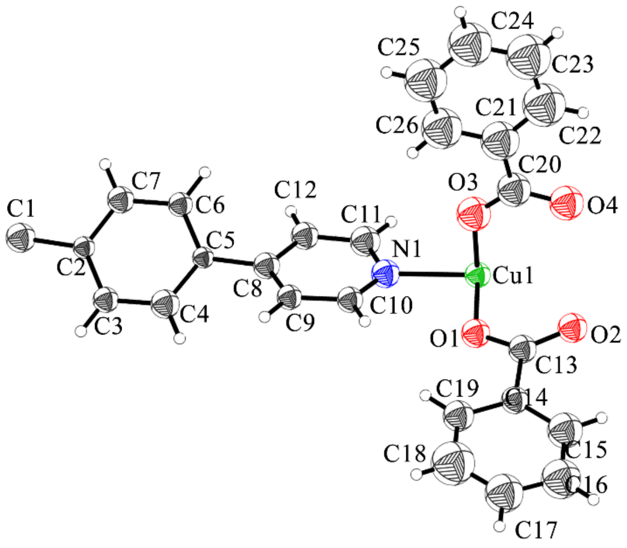

Figure S13. Ortep view of the asymmetric unit of **TPPM-CPW(Ph)•BnOH**. Probability level 30%. The structural model only shows the atoms belonging to the framework. It was not possible to model the BnOH molecules, but only to observe their potential in the cavities (see Figure S15).

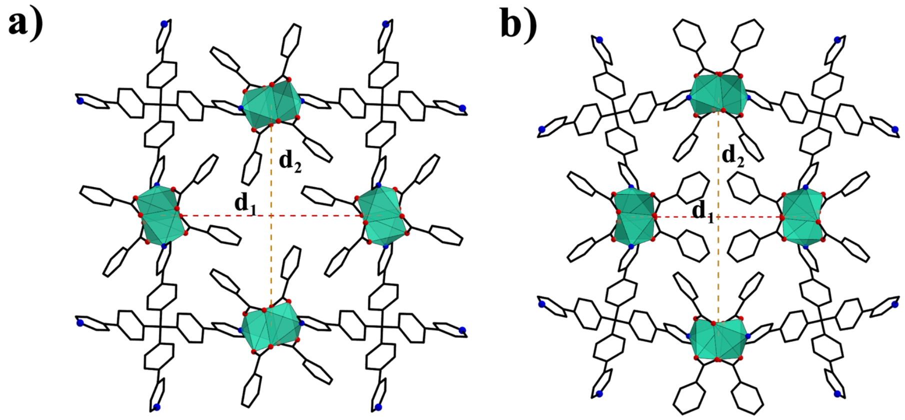

Figure S14. Comparison of the SBUs centroids distances in **TPPM-CPW(Ph)•BnOH** and **TPPM-CPW(Ph)** empty phase, both oriented along the *c*-axis. The distances between the centroids are depicted as red ($\mathbf{d_1}$) and orange ($\mathbf{d_2}$) dashed lines. $\mathbf{d_1}$(a) = $\mathbf{d_2}$(a) = 16.294(3) Å, $\mathbf{d_1}$(b) = 13.202(3) Å; $\mathbf{d_2}$(b) = 18.015(5) Å. Hydrogen atoms have been omitted for clarity.

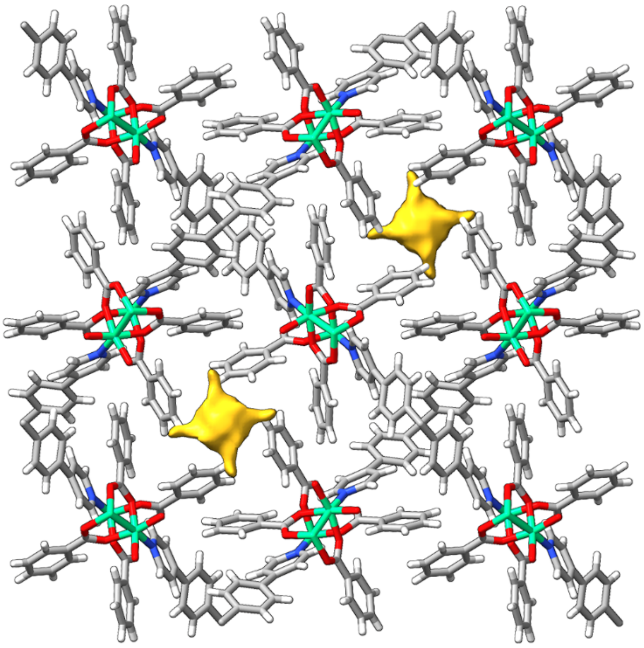

Figure S15. Difference Fourier map calculated on the kinematically-refined **TPPM-CPW(Ph)•BnOH** structural model. The electrostatic potential is reported as a yellow surface (isosurface level 3σ[ΔV(r)]), with the copper atoms in green, nitrogen atoms in blue, carbon atoms in light grey and hydrogen atoms in white.

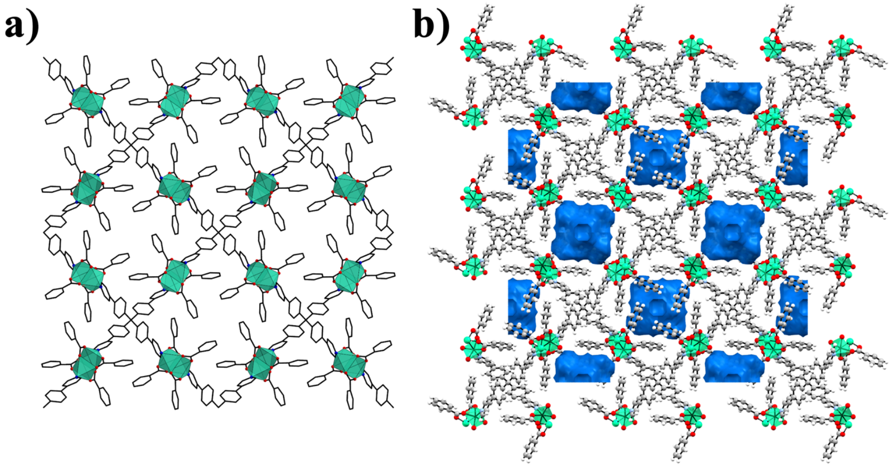

Figure S16. Crystal structure expansion of **TPPM-CPW(Ph)•BnOH** oriented along the crystallographic *c*-axis (a) with its relative isolated voids as blue surfaces (b).

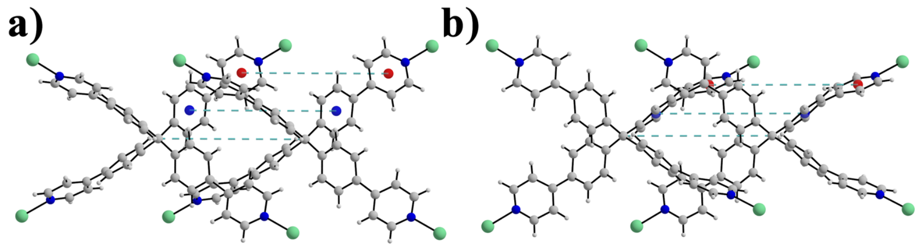

Figure S17. View of the principal inter-framework distances along the *c*-axis for the two **TPPM** fragments in the asymmetric unit of **TPPM-CPW(Ph)•BnOH** (a) and of the **TPPM-CPW(Ph)** empty phase (b). The rings centroids of the pyridyl and phenyl rings are represented in red and blue, respectively. The three distances (turquoise dashed lines) are equal for both fragments and equivalent to 8.303(3) Å for **TPPM-CPW(Ph)•BnOH** and 9.005(4) Å for the **TPPM-CPW(Ph)** empty phase, respectively.

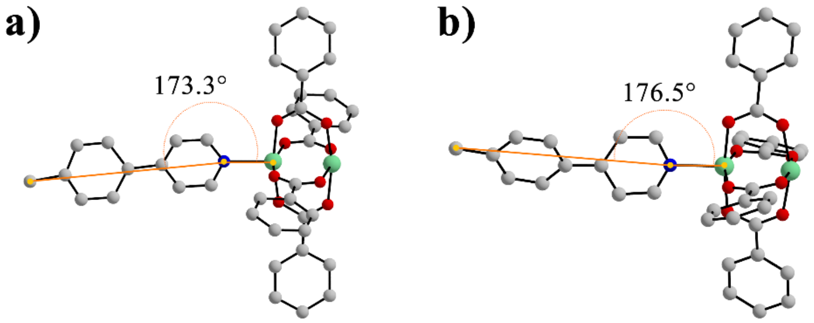

Figure S18. Asymmetric unit of **TPPM-CPW(Ph)•BnOH** (a) and of the **TPPM-CPW(Ph)** empty phase (b). The angle C1-N1-Cu1 highlights the differences in terms of coordination geometry between both crystal structures.

2.3 *Powder X-ray Diffraction (PXRD)*

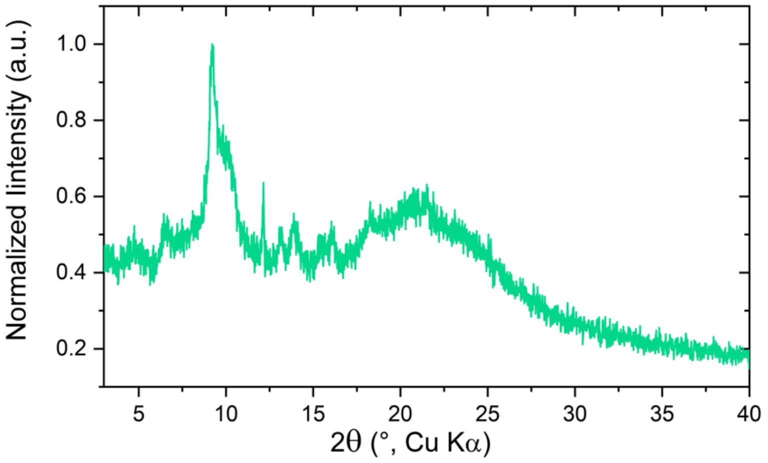

Figure S19. PXRD profile of **TPPM-CPW(Me)** after the 2 hours of exposure at atmospheric conditions.

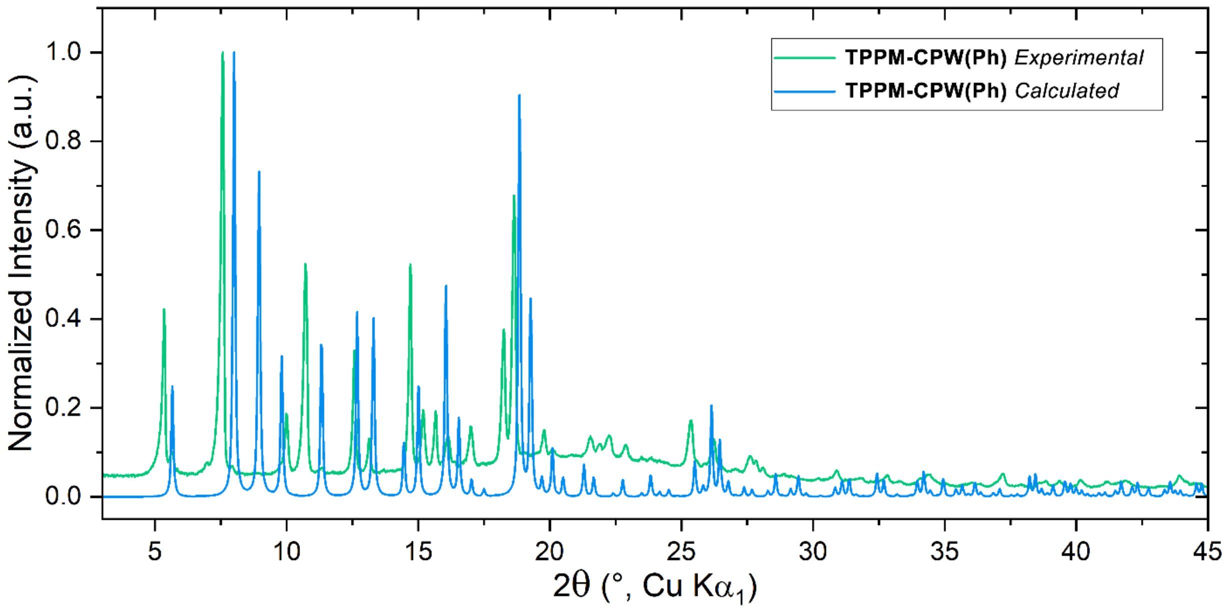

Figure S20. Comparison between the experimental and calculated PXRD profile of **TPPM-CPW(Ph)**. The calculated diffractogram of **TPPM-CPW(Ph)** corresponds to its empty phase, since it does not contain solvent molecules trapped in its framework.

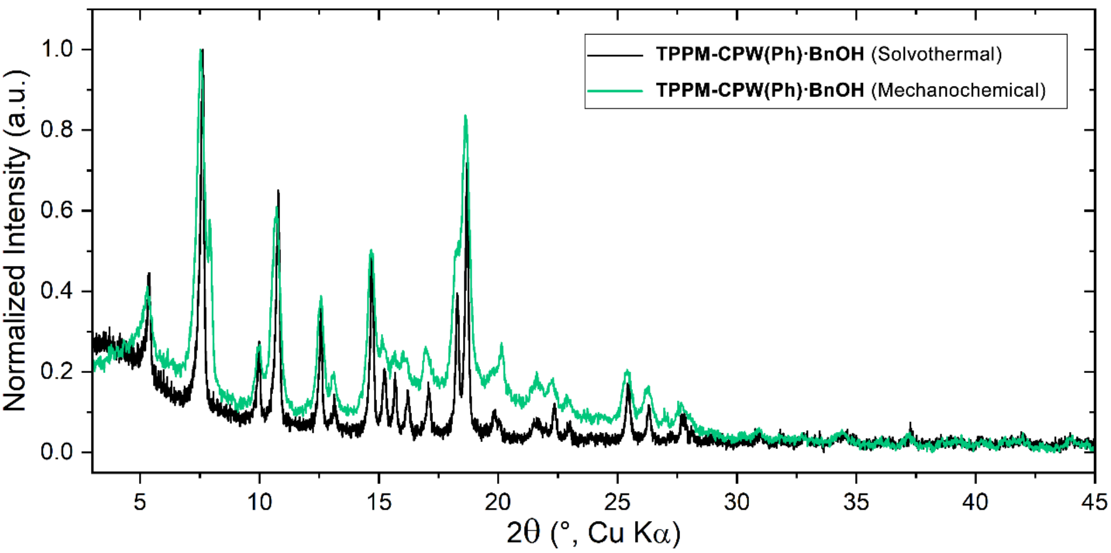

Figure S21. Superposition of the PXRD profiles of the **TPPM-CPW(Ph)•BnOH** crystals obtained through soaking of the solvotermal product (black trace) and mechanosynthesis (green trace).

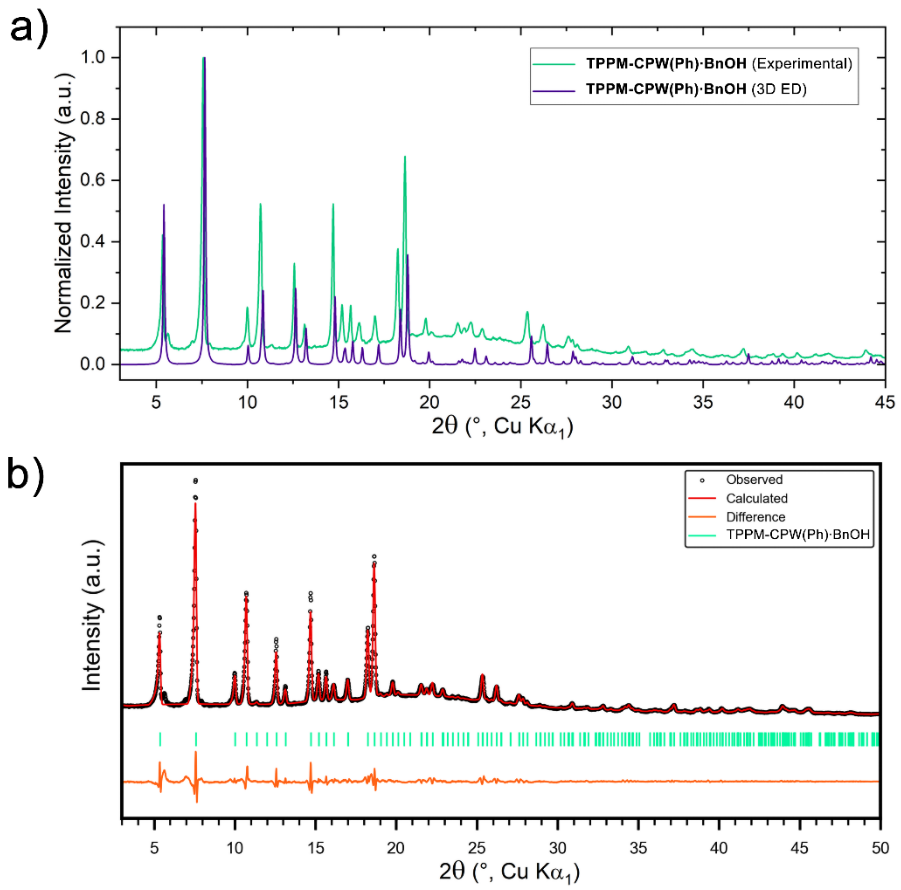

Figure S22. PXRD profile comparison between the experimental and calculated patterns of **TPPM-CPW(Ph)•BnOH** (a). Profile fit from Le Bail refinement on **TPPM-CPW(Ph)•BnOH**. The range is shown for 2θ values of 3-50 for clarity, whereas the refinement was carried out in the range 3-94°. The refinement converged to Rp = 3.39%, wRp = 6.05% and GOF = 2.29.

Table S4. Comparison between the unit cell parameters of **TPPM-CPW(Ph)•BnOH** obtained from 3D ED analysis and from the Le Bail refinement on PXRD data.

	3D ED	PXRD
a (Å)	23.043(5)	23.2663(8)
b (Å)	23.043(5)	23.2663(8)
c (Å)	8.803(2)	8.8096(5)
α (°)	90	90
β (°)	90	90
γ (°)	90	90
Vol (Å^3)	4674.2(3)	4768.8(6)

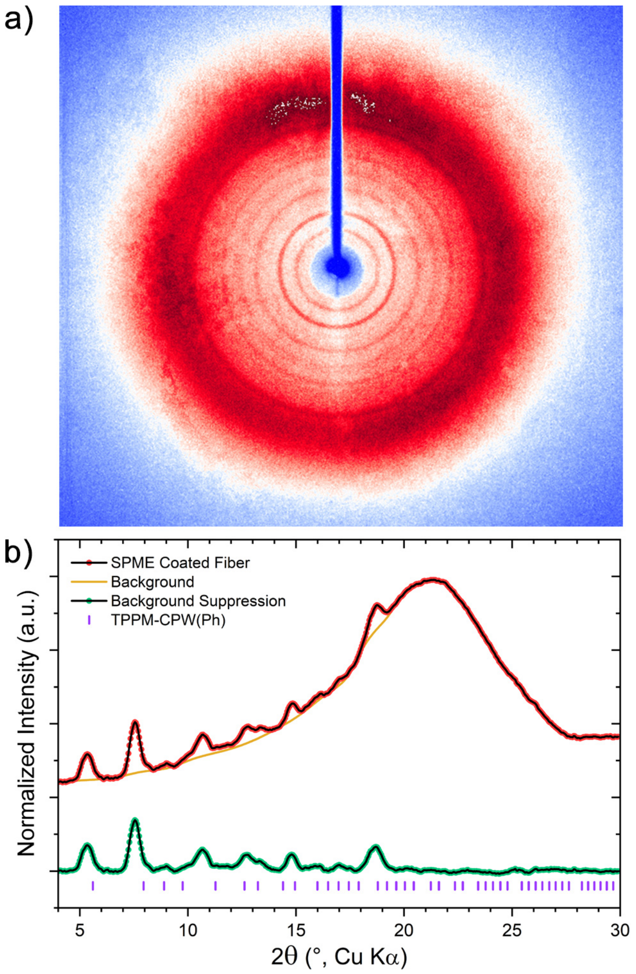

Figure S23. X-ray diffraction analysis on the SPME fiber coated with **TPPM-CPW(Ph)** after the desorption in GC injection port at 250 °C in He atmosphere. (a) Diffraction pattern of the fiber collected with an area detector. (b) PXRD diffractogram calculated from the 2D pattern. The amorphous profile has been interpolated as background and subtracted from the experimental pattern, which simplifies the visualization of the peaks correlated to the **TPPM-CPW(Ph)** phase, as also highlighted by their calculated positions.

3 Thermogravimetric Analyses (TGA)

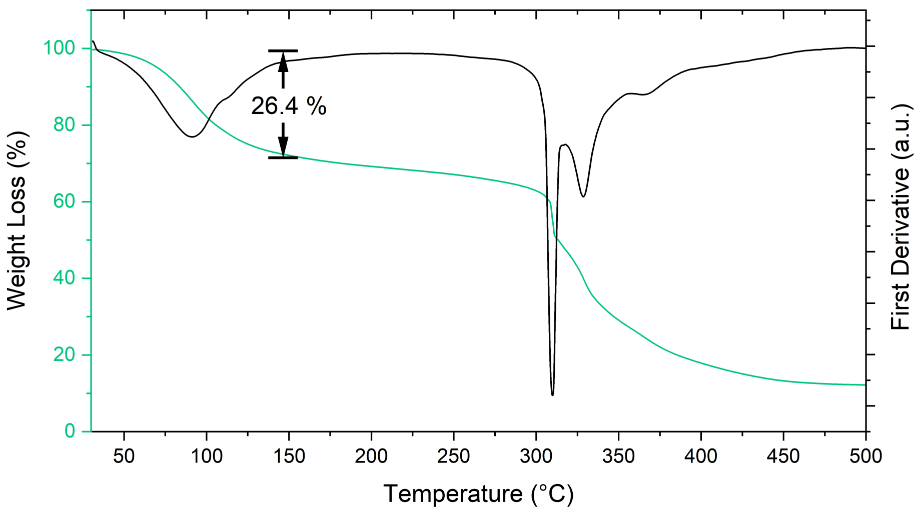

Figure S24. Thermogravimetric path recorded on the **TPPM-CPW(Ph)•BnOH** crystal phase.

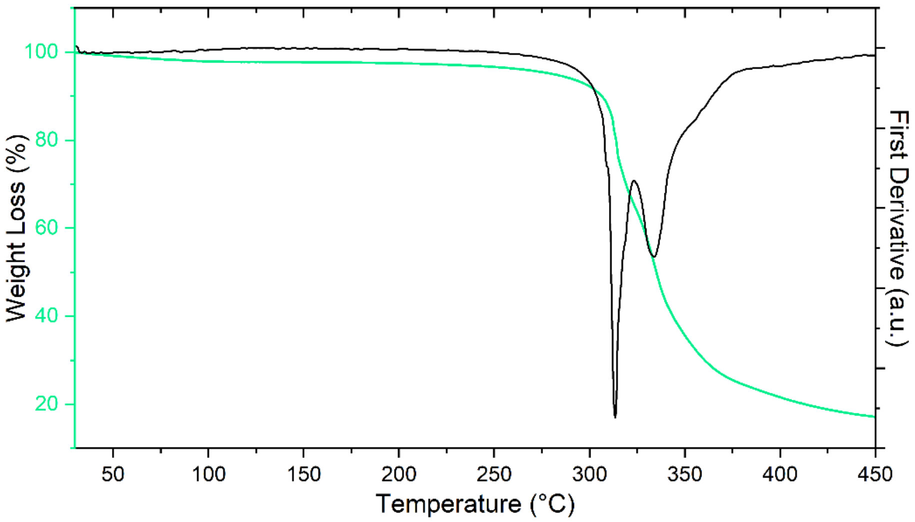

Figure S25. Thermogravimetric path recorded on the **TPPM-CPW(Ph)** crystal phase.

Relation adopted for the solvate stoichiometry (SS^{TGA}) calculation from TGA data:

$$SS^{TGA} = \frac{MW^{Host}}{MW^{Guest}} \frac{\Delta W(\%)}{100 - \Delta W(\%)}$$

where $\Delta W(\%)$ is the measured weight loss percentage, while MW^{Host} and MW^{Guest} are the molecular weight of the **TPPM-CPW(Ph)** MOF repeating unit and of BnOH, respectively.

4 NMR Characterization

The ^{1}H NMR spectra were collected in DMSO-d_6 with a few drops of DCl/H_2O 30% v/v to solubilize the framework completely. Moreover, the acidic environment reduces the ligand affinity toward the Cu(II) centers hampering their paramagnetic influence over the ligand chemical shift. The measurements have been carried out on a JEOL 600MHz ECZ600R.

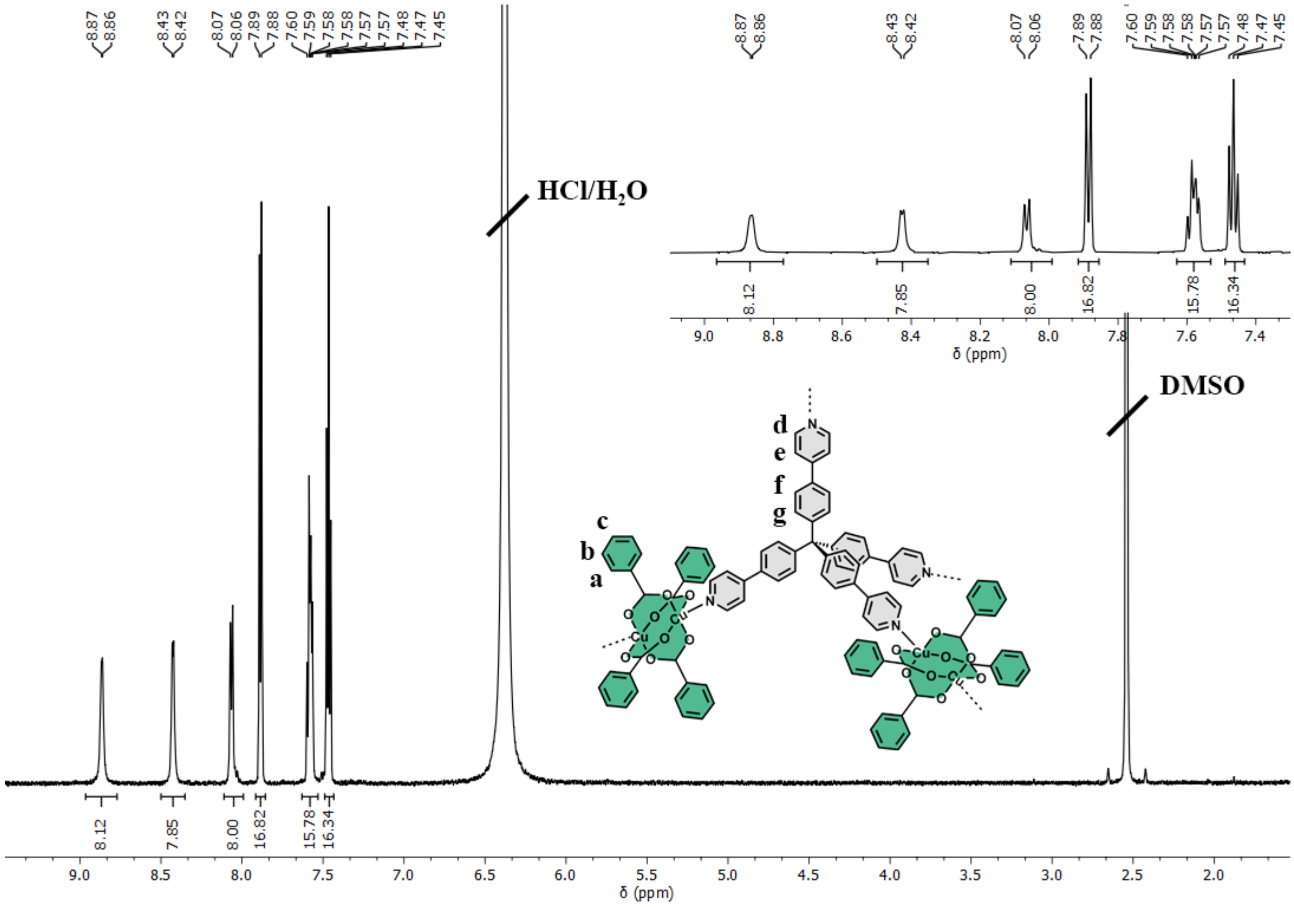

Figure S26. ^{1}H NMR spectrum of **TPPM-CPW(Ph)** (600 MHz, DMSO-d_6) d (ppm). 8.86 (m, 8H, d), 8.43 (d, J = 5.2 Hz, 8H, e), 8.06 (d, J = 8.2 Hz, 8H, f), 7.89 (d, J = 7.5 Hz, 16H, a), 7.58 (m, 16H, c - g), 7.47 (t, J = 7.6 Hz, 16H, b).

5 Gas sorption measurements

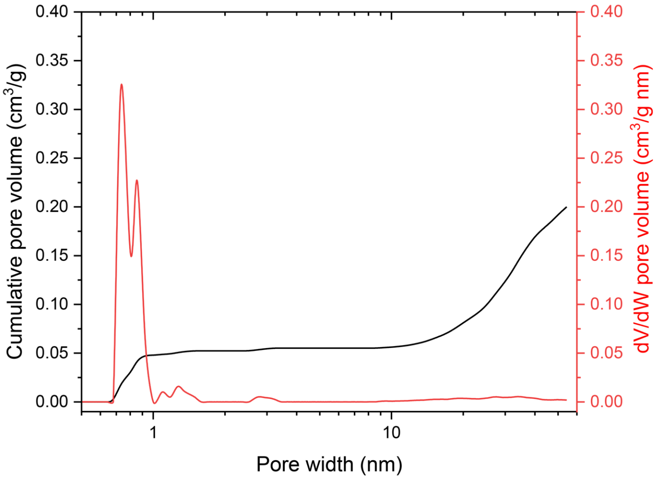

Figure S27. Pore size distribution and cumulative pore volume obtained from N_2 adsorption isotherms at 77 K for **TPPM-CPW(Ph)**.

Table S5. Textural parameters obtained from N_2 adsorption isotherms at 77 K for **TPPM-CPW(Ph)**.

	**TPPM-CPW(Ph)**
SSA_{BET} (m^2 g^{-1})	206
$SSA_{Langmuir}$ (m^2 g^{-1})	260
Pore volume (cm^3 g^{-1})	0.29
Peak pore size (nm)	0.74, 0.85

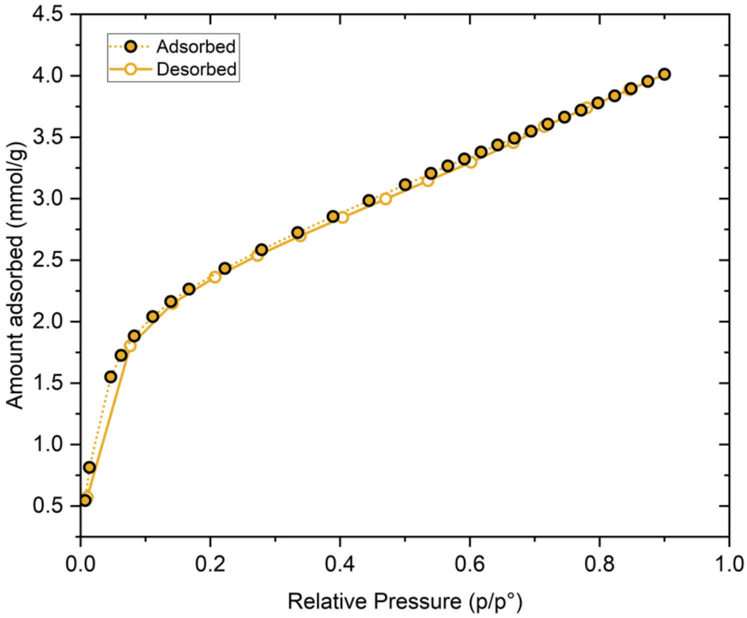

Figure S28. CO_2 adsorption isotherm of **TPPM-CPW(Ph)** measured at 195 K.

6 QM Calculations

The electrostatic potential running along the cavities of **TPPM-CPW(Me)** and **TPPM-CPW(Ph)** was calculated using the Material Studio software suite.[1] The calculation was carried out with the DMol3 package with a GGA-PBE functional, considering the crystal symmetry and unrestricted spin. The calculated potential was subsequently displayed over the electron density map (isovalue 0.2).

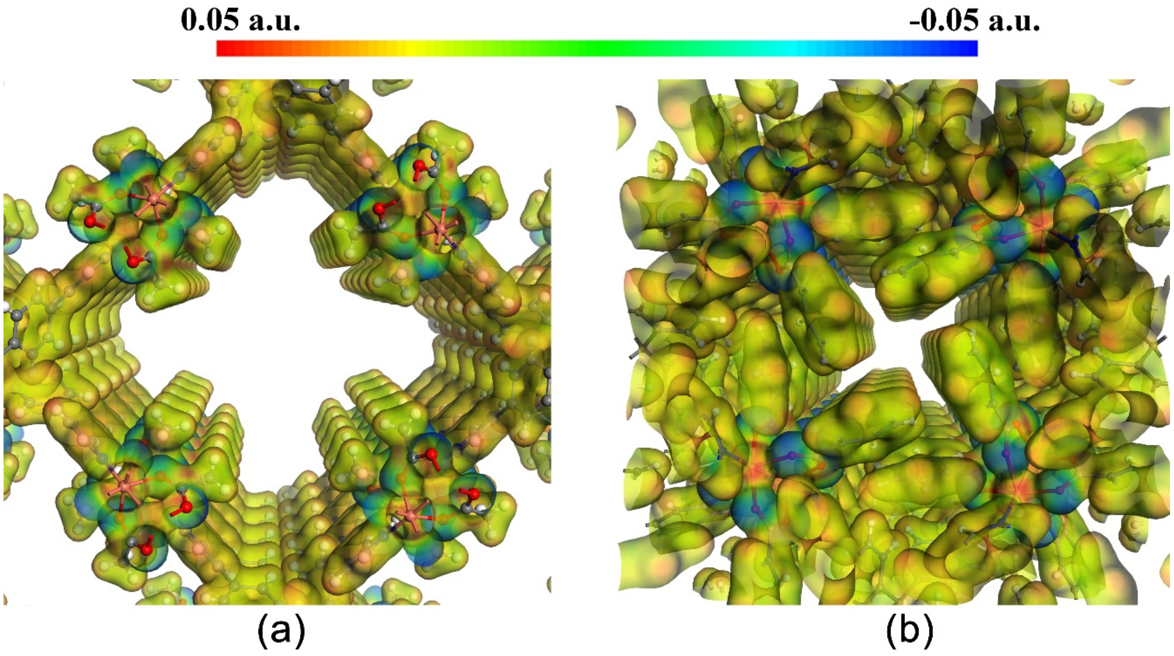

Figure S29. Potential electrostatic surfaces of the expanded structure for **TPPM-CPW(Me)** (a) and **TPPM-CPW(Ph)** (b). The expanded structures have been oriented along the crystallographic *c*-axis, to emphasize the potential distribution along their channels.

(1) Meunier, M.; Robertson, S. *Materials Studio* 20th Anniversary. *Molecular Simulation* **2021**, *47* (7), 537–539. https://doi.org/10.1080/08927022.2021.1892093.

7 SPME-GC-MS analysis of fluorinated anaesthetics

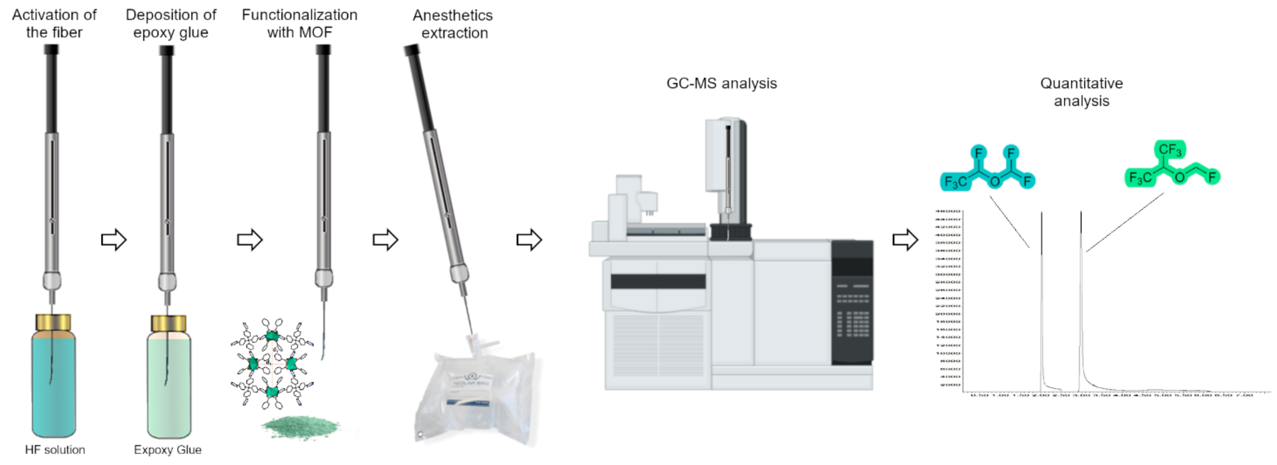

Figure S30. Scheme describing the adopted analytical procedure.

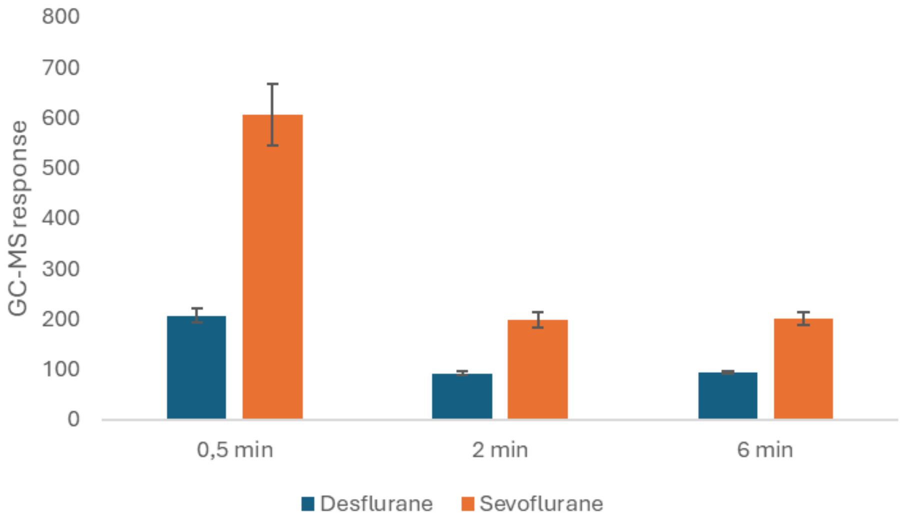

Figure S31. SPME-GC-MS analysis of desflurane and sevoflurane (each at 500 ppb_v) in ambient air using the **TPPM-CPW(Ph)** fiber: evaluation of the extraction time (n = 5).
